# A Polarizable Atomic Multipole-Based Force Field for Molecular Dynamics Simulations of Anionic Lipids

**DOI:** 10.3390/molecules23010077

**Published:** 2017-12-31

**Authors:** Huiying Chu, Xiangda Peng, Yan Li, Yuebin Zhang, Guohui Li

**Affiliations:** 1 Laboratory of Molecular Modeling and Design, State Key Laboratory of Molecular Reaction Dynamics, Dalian Institute of Chemical Physics, Chinese Academy of Science, 457 Zhongshan Road, Dalian 116023, China; chuhy2009@dicp.ac.cn (H.C.); pengxd@dicp.ac.cn (X.P.); liyan1982@dicp.ac.cn (Y.L.); zhangyb@dicp.ac.cn (Y.Z.); 2 Chinese Academy of Science, University of Chinese Academy Sciences, Beijing 100049, China

**Keywords:** Lipid polarizable force field, DMPG, POPS

## Abstract

In all of the classical force fields, electrostatic interaction is simply treated and explicit electronic polarizability is neglected. The condensed-phase polarization, relative to the gas-phase charge distributions, is commonly accounted for in an average way by increasing the atomic charges, which remain fixed throughout simulations. Based on the lipid polarizable force field DMPC and following the same framework as Atomic Multipole Optimized Energetics for BiomoleculAr (AMOEBA) simulation, the present effort expands the force field to new anionic lipid models, in which the new lipids contain DMPG and POPS. The parameters are compatible with the AMOEBA force field, which includes water, ions, proteins, etc. The charge distribution of each atom is represented by the permanent atomic monopole, dipole and quadrupole moments, which are derived from the ab initio gas phase calculations. Many-body polarization including the inter- and intramolecular polarization is modeled in a consistent manner with distributed atomic polarizabilities. Molecular dynamics simulations of the two aqueous DMPG and POPS membrane bilayer systems, consisting of 72 lipids with water molecules, were then carried out to validate the force field parameters. Membrane width, area per lipid, volume per lipid, deuterium order parameters, electron density profile, electrostatic potential difference between the center of the bilayer and water are all calculated, and compared with limited experimental data.

## 1. Introduction

Biological membranes are indispensable components of cells. The main function of membranes is to maintain the mechanical and chemical integrity of cell by controlling the flow of molecules and signals in and out of the cell. Membranes are mostly made of phospholipids, while membrane proteins, cholesterols, ions, and water etc. are also integral parts of membranes [1]. Understanding of the structural and dynamic properties of phospholipid bilayers at the atomistic level is fundamentally important. Molecular modeling and simulations are widely used in the study of the structure, function, and dynamics of membrane-bounded proteins. The classical all-atom force fields of lipid treat the electrostatic interactions with the fixed atomic charges. The classical force fields have been performed on many membrane systems. Simulations of phospholipids are performed on many phospholipids [2,3,4], which due to the fact that many protein properties are dependent on the lipid composition. Large membrane proteins are also investigated using classical all-atom force fields, such as ion channels [5,6,7], G-protein-coupled receptors, and outer membrane proteins.

Theoretical investigations have shown that the cluster polarizabilities and the dielectric constants of fluids, which determined the condensed-phase environment, significantly affect the polarizability and conformation of a solvated molecule. The polar headgroups of lipids face the high dielectricity of water on one side, and interface with the low-dielectric hydrocarbon core on the other side [8]. The electronic polarizations experienced exterior and interior of membrane by an embedded molecule are very different [9,10]. If a molecule stays within the membrane, its behavior may be different from the same molecule on the surface of bilayer due to the different polarization environment [11,12]. Roux et al. [13] have investigated the ion selectivity of several membrane-binding channels and transporters, their results showed that although the fundamental physical properties could be described using the nonpolarizable models, more detailed understanding of the conformation-driven super-selectivity depends on improvements in force field models considering explicit polarizability.

Efforts have been made to develop polarizable force fields of lipids. Patel and co-workers have developed a polarizable force field for dimirystoylphosphatidylcholine (DMPC) and dipalmityol-phosphatidylcholine (DPPC) based on the charge equilibration (CHEQ) force field approach [14,15]. The CHEQ force field has been applied to the studies of bilayers and monolayers of lipid, as well as membrane-bounded protein channels, such as gramicidin A [16]. Taking the water permeation for example [15], the simulations using the polarizable force field showed higher permeation than the results with non-polarizable models. It was suggested that fixed-charge force field could not produce the expected dielectric property of the nonpolar hydrocarbon region, and water molecules in membrane interior have large dipole moments similar to the waters in the bulk [17]. Drude oscillator- based polarizable force field for lipids has also been developed [18,19]. More recently Drude force fields of a series zwitterionic lipids [8,20], cholesterol [21], and sphingomyelin [21] have been established. In all the simulations using Drude force field, the description of the membrane dipole potential has been improved, as a result of the inclusion of atomic polarizabilities.

The AMOEBA polarizable force field utilizes the induced dipole model to describe the intra- and intermolecular polarization effect. In this model, a point dipole is induced at each contributing center (e.g., an atom) in response to the total electric field from the surroundings. The total field is determined self-consistently via an iterative procedure that minimizes the polarization energy or by means of the extended Lagrangian method in MD simulations [22,23]. In addition to the polarization effect, another limitation of fixed-point charge force fields is that the atomic charge-based representation of permanent electrostatics is inadequate unless additional off-center partial charges are added [24]. AMOEBA employs atomic monopole, dipole and quadrupole moments to improve the permanent electrostatic representation [25,26,27]. The incorporation of higher order atomic multipoles showed great improvement of the quality of electrostatic potential [25,26], protein-ligand binding [28,29,30,31,32], and crystal structure predictions for simple organic molecules [33,34,35]. The free energy profiles of K^+^ and Na^+^ permeating through the gramicidin A channel embedded in DMPC are characterized by our group with using the AMOEBA polarizable force field by our group [36]. The previous calculated potential mean forces (PMF) for K^+^ permeation using the additive force field demonstrated an unexpectedly high free energy barrier of 10–20 kcal/mol [18,37,38,39,40,41,42,43,44,45,46,47,48], whereas the experimental data indicated that the K^+^ permeates through the gA channel with a small free energy barrier [49,50]. Compared with the results produced by additive force field, the free energy barriers for K^+^/Na^+^ through gA channel are significantly decreased [36], which are depended on the explicitly described polarization effects in AMOEBA. The free energy barrier of K^+^ is 4.8 kcal/mol, and Na^+^ is 5.6 kcal/mol, which are in agreement with the experimental selectivity of gA channel [50], and better than additive force fields.

In this work, we present the development of the new anionic phospholipid dimyristoyl phosphatidylglycerol (DMPG) and 1-palmitoyl-2-oleoyl-3-*sn*-phosphatidylserine (POPS) force field based on polarizable multipole electrostatic representation of the AMOEBA framework. The permanent atomic multipoles were obtained from ab initio calculations on small fragments that make up the lipid molecule. The combine Distributed Multipole Analysis (DMA) [51] and electrostatic potential fitting were applied to derive conformation-independent multipoles [27]. The valence and van der Waals parameters have been derived from liquid simulations of small organic molecules [52] containing similar functional groups. Merging of inter- and intramolecular interactions at short distances separation between fragments, which include electrostatics, vdW, and torsional contribution, is determined via comparison to gas phase ab initio conformational and association energy for small fragments. The model is examined and validated by molecular dynamics simulations of the phospholipid bilayer in water and comparison with limited experimental data.

### Potential Energy Model

The AMOEBA force field is a polarizable molecular mechanics model that treats electrostatic interactions with higher order moments up to quadrupoles. The potential energy model has been described previously [27,53,54], and is briefly explained here. The potential energy function comprises bonded and non-bonded interactions. The valence interactions include bond-stretching, angle-bending, bond-angle stretch-bending, out-of-plane bending, and torsion interactions. Non-bonded interactions include van der Waals, permanent and induced electrostatics. The total energy of the system is described by:

(1) $$U=U_{bond}+U_{angle}+U_{b\theta }+U_{oop}+U_{torsion}+U_{vdW}+U_{ele}^{perm}+U_{ele}^{ind}$$

**Figure 1:**
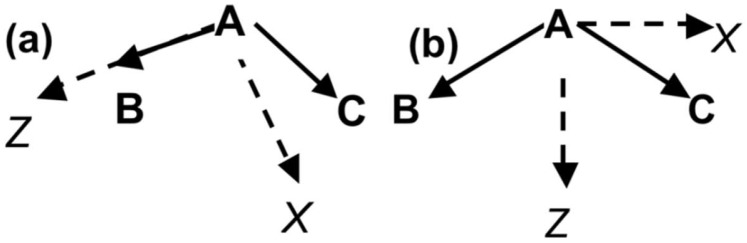
Local frame definition for the DMPG and POPS models. (**a**) Local frame definition of *Z*-then-*X*, where the first bonded atom defines the *Z*-axis, and the second bonded atom defines the *ZX*-plane and the positive *X*-direction; (**b**) local frame definition of Bisector, where the bisector of two neighboring atom defines the *Z*-axis.

Bonded interactions in the AMOEBA force field adopt non-harmonic functional forms. Bond stretching energies are expressed by the fourth-order Taylor expansion of the Morse potential. A sixth-order potential and a six-term Fourier series expansion are used for angle bending and torsion terms, respectively. Additionally, out-of-plane bending was restrained at sp^2^-hybridized trigonal centers with a Wilson-Decius-Cross function. The repulsion-dispersion vdW interaction in AMOEBA is modeled by the buffered 14-7 potential [55]. Reduction factors are added to hydrogen atoms to move the vdW interaction center (not the mass) closer to the heavy atoms they are connected to. Permanent electrostatic interaction energies and forces are calculated through the multipole-multipole interaction. Multipoles are defined at atomic centers in relation to a local frame defined by other bonded atoms. Figure 1 shows examples of the local frame definition used in the DMPG and POPS models, including the “*Z*-then-*X*” and Bisector conventions. Polarizable atomic dipoles are calculated in AMOEBA to describe the polarization effect [56] using the Thole iterative induction model [57]. A damped polarization interaction was imposed at a very short range in order to avoid a well-known artifact of point polarizability models by smearing one of the atomic multipole moments in each pair of interaction sites.

OpenMM [19] offers accelerated GPU-based calculations. In this work, the multiple time step (MTS) algorithm is used in all AMOEBA molecular dynamics simulations, in which nonbonded interactions including polarization are updated every 2 fs.

## 2. Results and Discussion

To validate the quality of our model, we have systematically examined a series of physical properties of lipids in gas and solution phase. The electrostatic properties in the gas phase and the torsional conformation energies were first inspected. The important advantage of the AMOEBA polarizable force field is that the electrostatic properties in both gas and solution phases can be captured.

### 2.1. Electrostatic Properties of Headgroup

**Figure 2:**
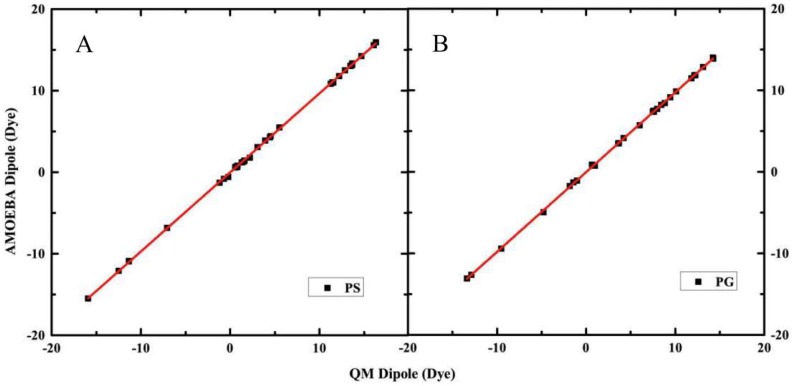
Comparison of headgroup molecular dipole *x*-, *y*-, *z*-components predict by AMOEBA and QM (MP2/6-311++G(2d,2p)) for headgroups. The AMOEBA permanent atomic multipole was obtained from four different conformations and validated on additional 6 conformations. (**A**) Comparison of headgroup molecular dipole *x*-, *y*-, *z*-components predict by AMOEBA and QM for PS headgroup; (**B**) Comparison of headgroup molecular dipole *x*-, *y*-, *z*-components predict by AMOEBA and QM for PG headgroup.

It is also essential to ensure the electrostatic parameters suitable for most different conformations of the headgroup, which means the transferability of the electrostatic properties. The electrostatic properties of headgroup are validated using the comparison of the total of dipole and the *x*-, *y*-, *z*-components moment, and the electrostatic potential on grids around the headgroup. A total of 6 conformations of PG and PS headgroups were selected as the validation set, and these structures were different from the four conformations of “training” set used in the parameterization. In Figure 2, the comparison of headgroup molecular dipole *x*-, *y*-, *z*-components predicted by AMOEBA against QM (MP2/6-311++G(2d,2p)) is presented. Figure 2 shows that the total and *xyz* components of the headgroup dipole moment are well reproduced in the different conformations using the AMOEBA parameters with a correlation coefficient (R^2^) of 0.999 for both PG and PS headgroup, and AMOEBA shows a perfect slope 0.97 for PS headgroup, and 0.98 for PG headgroup. The intercept is only −0.03 dye for PS, and −0.04 dye for PG headgroup. The standard and unsigned average errors are 0.11 and 0.28 Dye for PS, 0.25 and 0.48 dye for PG headgroup predicted by AMOEBA.

Because of the charged nature of the headgroups, it is not surprising that the dipole moments are fairly large and easy to capture. Thus it is a somewhat necessary but not sufficient measurement of the quality of electrostatic parameters. We have further investigated the detailed electrostatic potential distribution around the headgroups. The average RMSE of the calculated electrostatic potential is calculated for each force field and significant variation has been observed. The unsigned RMSE of AMOEBA ESP is only 0.36 kcal/mol/e on a grid surrounding the PS headgroup for the ten conformations, and for the PG group the RMSE is only 0.33 kcal/mol/e, which is likely very close to the uncertainty of the ab initio method we are comparing against (MP2/6-311++G(2d,2p)). Due to the intramolecular polarization model in AMOEBA force field, the transferability of the small model electrostatic multipoles of AMOEBA is quite satisfactory. The test of the electrostatic potential on a grid surrounding the group is very important and necessary for the electrostatics properties of force field before other components validation. One advantage of AMOEBA is that the gas phase electrostatic properties can be rigorously compared with QM ab initio results, and the polarizable force field can be transferred from gas phase to solution.

### 2.2. Modification of Torsion Parameters

**Figure 3:**
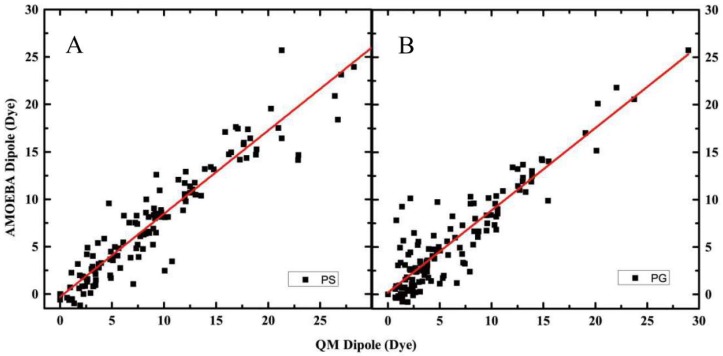
Comparison of torsional conformation energies predict by AMOEBA and QM (MP2/6-31G(d,p)). (**A**) Comparison of torsional conformation energies predict by AMOEBA for PS headgroup and QM; (**B**) Comparison of torsional conformation energies predict by AMOEBA for PG headgroup and QM.

The dihedral angle was scanned by minimizing molecules about the rotatable bond at 30° intervals with restraints and calculated at the MP2/6-31G(d,p) level. The two head group molecules were fitted to the QM data based on the existing electrostatic parameters. In total 12 torsions of the PS group and 13 torsions of the PG group were scanned, with each rotatable bond was minimized for 11 conformations, and the comparison of torsional conformation energies predict by AMOEBA against QM (MP2/6-31G(d,p)) is shown in Figure 3 and Table S1. The correlation coefficients are 0.92 and 0.87 for PS and PG headgroup, respectively. The standard error and unsigned average error compared with QM values are 7.12 and 1.19 kcal/mol for PS headgroup, which are a little worse than those values for PG headgroup (2.27 and 1.27 kcal/mol). PS headgroup and PG headgroup both produced the same slope (0.88).

### 2.3. Validation of Interaction Headgroup with Small Molecules including Water and Ions 

**Figure 4:**
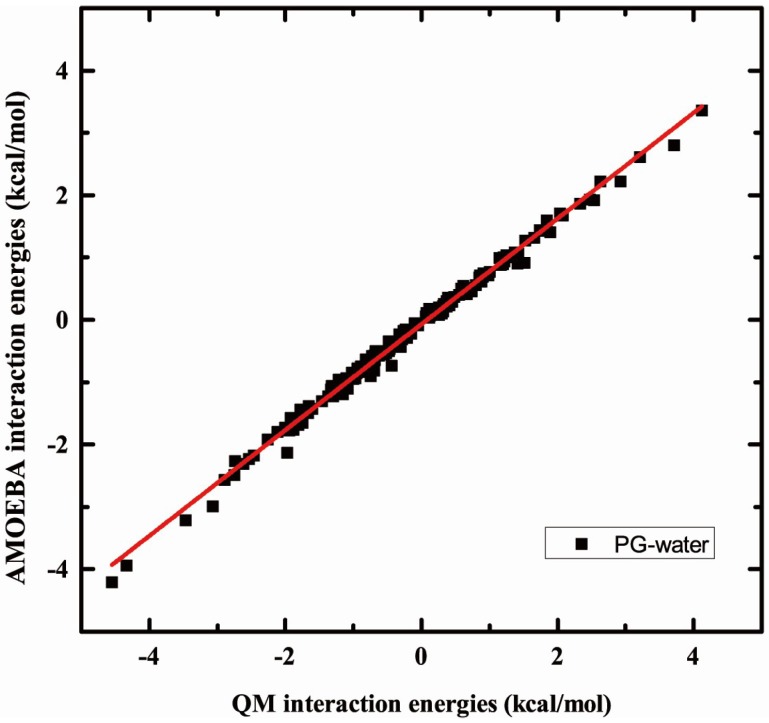
Comparison of association energies predict by AMOEBA and QM (MP2/6-311++G**). The binding energies were calculated for the PG headgroup with water.

Due to the penetrability of water along the membrane, it is important to accurately describe the interaction between the water molecule and the headgroup accurately. The results of water interactions with tails have been shown in previous papers. Taking the dimer of the PG headgroup with water, and the PS headgroup with Na^+^ for example, the interaction energies are calculated against with QM results. Approximately 170 different conformations of the PG headgroup with water, and about 20 different conformations of the PS headgroup with Na^+^ were used in this association energy calculation. The PG headgroup with water results are shown Figure 4. The correlation coefficient is 0.99, and the slope is 0.85, which means the interaction energies predicted by AMOEBA are a little lower than the QM results, but the standard error and unsigned average error compared with the QM values are only 0.06 and 0.18 kcal/mol. Therefore, although the interaction predicted by AMOEBA is lower than the QM result, the difference of energy values is quite acceptable. Another validation is performed on the comparison between interaction energies predicted by AMOEBA and QM of the PS headgroup with Na^+^, where approximately 20 conformations are considered in the comparison. The correlation coefficient is 0.998, which means the interaction energies predicted by AMOEBA a quite a fine match with the QM results.

### 2.4. Simulation and Validation

**Figure 5:**
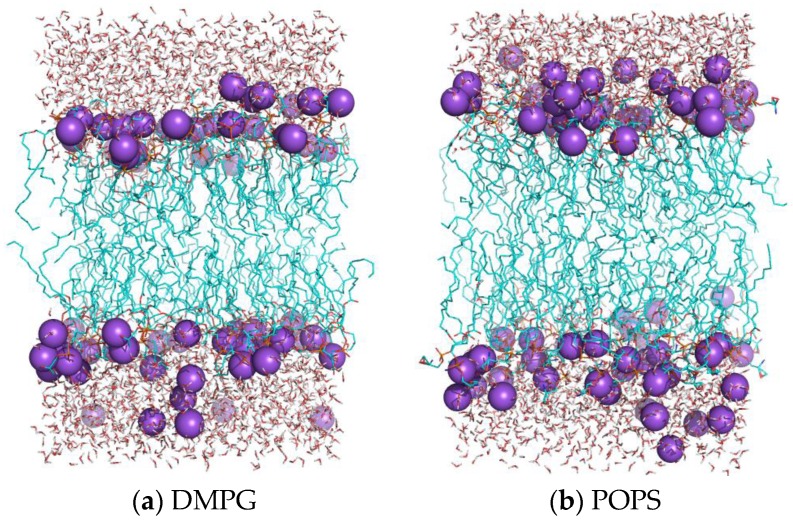
The structures of the DMPG (**a**); POPS (**b**) under tensionless situation after the production run.

Although gas-phase properties provide valuable insight and guidance into force field development, and the gas phase electrostatic properties can be comparable with the QM results. Then the validation of solution phase properties is performed on the molecular simulation based on the AMOEBA force field, as compared with the limited experimental data on DMPG and POPS membranes. The final structures of AMOEBA-simulated DMPG and POPS systems are shown in Figure 5.

### 2.5. Structure of Lipid Bilayers

The value of area per lipid is often used to monitor the equilibration of simulations and used to compare with experimental value for validation purpose. The number of experimental area per lipid values has been reported for DMPG liquid-crystalline 62.5 Å^2^ [58]. In simulations, the area per lipid for DMPG and POPS membrane system was calculated using the formula:

(2) $$A_{L}=\frac{L_{x}\times L_{y}}{n_{\mathrm{lipid}}/2}$$

where L*_x_* and L*_y_* are the length in *x*-dimension and *y*-dimension of the simulation box, and n_lipid_ is the number of molecules in the system. The values of DMPG and POPS area per lipid are shown in Table 1. The average values of area per lipid produced by AMOEBA simulations are approximately 60.05 ± 0.73 Å^2^ for DMPG system, and 60.93 ± 0.55 Å^2^ for POPS system. CHARMM force field produces the 63.30 Å^2^ for DMPG [59], and GROMOS force field produces 55 Å^2^ for POPS system [60]. These results for the two systems are all around the experimental value, and the errors are less than ~3% [58].

The volume per lipid was also calculated using the formula below:

(3) $$V_{L}=\frac{L_{x}\times L_{y}\times L_{z}-n_{w}V_{w}}{n_{\mathrm{lipid}}}$$

where L*_z_* is the length in *z*-dimension of the simulation box, n_w_ is the number of water molecules, and V_w_ is the volume of one water molecule, which was set to be 30.53 Å^3^ for the calculation. The values are shown in Table 1, and the average values of the volume per lipid are 1021.10 Å^3^ for DMPG system, 1130.94 Å^3^ for POPS system in the tensionless-simulating system. The difference between two separately simulation is only 0.32 Å^3^ for DMPG, 0.51 Å^3^ for POPS, which means the simulation is stable.

**Figure 6:**
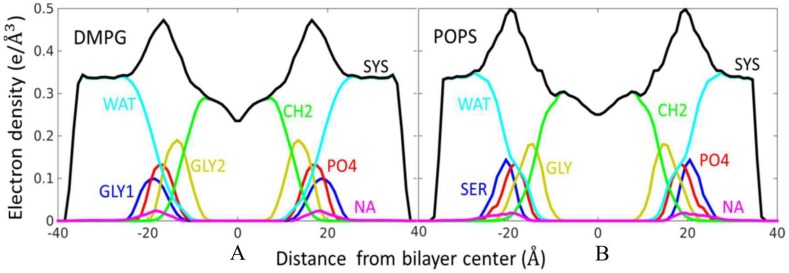
The total and decomposed electron density profiles of the lipid molecule. (**A**) The electron density profiles of the DMPG; (**B**) The electron density profiles of the POPS.

The electron density profiles (EDP) were calculated as first dividing the system into slabs along the bilayer normal, and summing the electron charges in each slab, then dividing by the slab volume. The electron charge is equal to the atomic number, minus the partial charge of the atom, without considering the atomic dipole and quadrupole contribution. The EDP for DMPG and POPS systems is shown in Figure 6. The EDP is decomposed into contributions from the following group: water, carbonyl glycerol groups (GLY, GLY1, GLY2 group), the phosphate (PO_4_ group), methylene unsaturated CH=CH terminal methyl (CH_2_). All of these profiles are symmetrical, and the results show that water penetrates up to carbonyl and glycerol group, and leaves the two tail hydrophobic methyl groups. From the profile of Na^+^, it should be noted that the peaks of Na^+^ are near the peaks of the phosphate group for both DMPG and POPS lipids, which means that the most Na^+^ ions locate near the phosphate group and interact with oxygen of phosphate group. From the EDP, the thickness of the DMPG and POPS bilayers can be calculated as the distance between the two peaks (see Table 1). The thicknesses of bilayers are 33.9 Å for DMPG system and 38.7 Å for the POPS system. The result of AMOEBA for DMPG is well agreement with experimental value, 33.8 Å [58]. From Figure 5, it can be seen that the Na^+^ almost located near the phosphate group, which is consistent with the EDP analysis.

**Figure 7:**
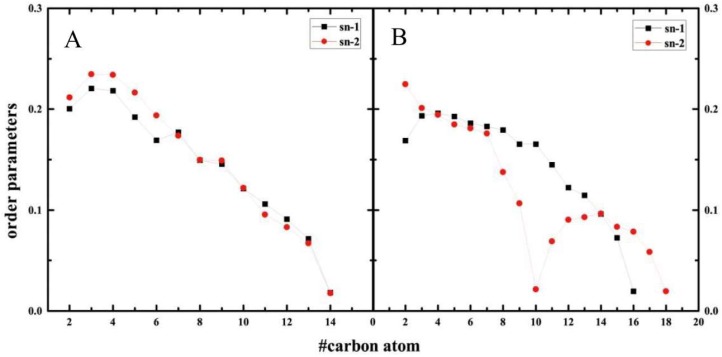
The order parameters for bilayer system averaged over the last 20 ns simulations. (**A**) The order parameters for DMPG bilayer system; (**B**) The order parameters for POPS bilayer system.

The dynamics behaviors of the DMPG and POPS tails are probed by measuring the relative orientation variation of deuterium connected with the carbon atoms of the alkyl chain. This parameter of orientation is named order parameters, S_CD_, and calculated with:

(4) $$S_{\mathrm{CD}}=\frac{1}{2}\langle 3\cos^{2}\theta -1\rangle$$

where θ is the angle between the vector C-D bond and the bilayer normal, and the 〈〉 represents the ensemble average. The simulated order parameters for the DMPG and POPS molecule systems are shown in Figure 7. When $S_{\mathrm{CD}}=0$, the hydrocarbon chains are random orientations or at an orientation of about 54° between vector and bilayer normal [20]. The simulated order parameters of the saturated lipid chain Sn-1 exhibit a decreasing profile. The unsaturated lipid chain Sn-2 of POPS contains one double bond between the C9 and C10, and the S_CD_ values of C9 and C10 in Sn-2 show a distinctive drop, which due to the *cis* double bond.

**Figure 8:**
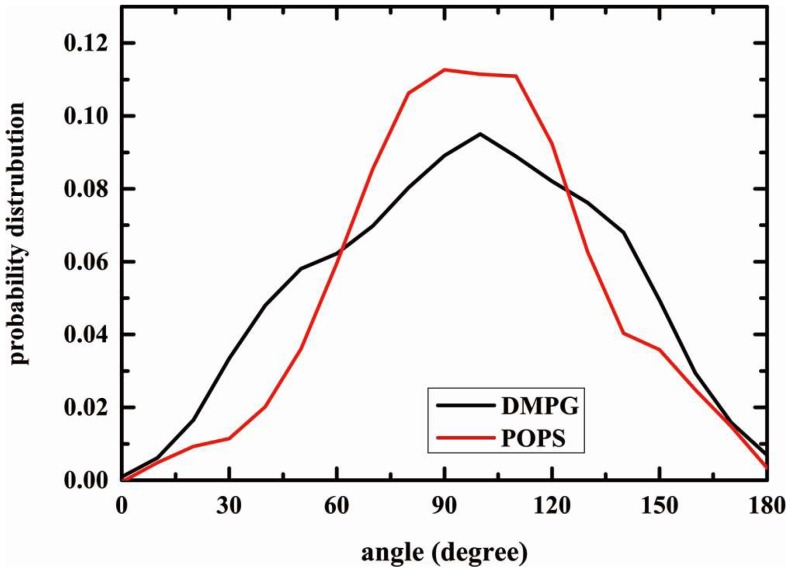
Probability distribution function of the angle between the lipid head P-N vector and the bilayer normal of the DOPC and POPE bilayer system from the last 20 ns.

The orientation of the DMPG and POPS headgroups is analyzed by measuring the angle between the phosphrous-C2 of glycerol for DMPG, and phosphrous-nitrogen vector and the bilayer normal for POPS. The probability distribution of the angle is plotted in Figure 8. The importance of the orientation in modulating the hydration of interfacial region has been discussed [61]. The most probable angle from AMOEBA simulations are about 90° of DMPG system and 100° of POPS system, in comparison with the experimental order [62]. The probability distribution of POPS is also narrower than those observed in DMPG, which suggesting a more restricted range of rotation angle for phosphate and serine group.

### 2.6. Dipole Potential 

The dipole potential of membrane remains a physical quantity, which is difficult to capture in molecular dynamics. The electrostatic potential difference is easy to be measured in experiment [63]. In molecular simulation, it is difficult to define the interfacial as the experimental measure. In this work, the electrostatic potential is calculated by converting the definition of induced dipole in AMOEBA. The induced dipole at each atomic site is computed as:

(5) $$\mu_{i,\alpha }^{\mathrm{ind}}=\alpha_{i}E_{i,\alpha }$$

(6) $$\mu_{i,\alpha }^{\mathrm{ind}}=\alpha_{i}\left(\underset{j}{\sum }T_{\alpha }^{\mathrm{ij}}M_{j}+\underset{j^{\prime }}{\sum }T_{\alpha }^{ij^{\prime }}\mu_{j^{\prime },\beta }^{\mathrm{ind}}\right)$$

where $E_{i,\alpha }$ is the sum of the fields generated by permanent multipoles $M=\left[q,\mu_{1},\mu_{2},\mu_{3},Q_{11},Q_{12},\ldots ,Q_{33}\right]$ and induced dipoles, and $\alpha_{i}$ is the atomic polarizability.

The electrostatic potential can be calculated as:

(7) $$\varphi_{z}=\int_{0}^{z}E_{z}\mathrm{dz}$$

The E_z_ is the average field which was calculated as the sum of each atom $E_{i,\alpha }$ in dividing slabs and then dividing by the number of atoms slab. It can be described below:

(8) $$E_{z}=\langle \delta \left(z-z_{m}\right)\underset{i}{\sum }\frac{\mu_{i,m}^{\mathrm{ind}}}{\alpha_{i}}\rangle$$

**Figure 9:**
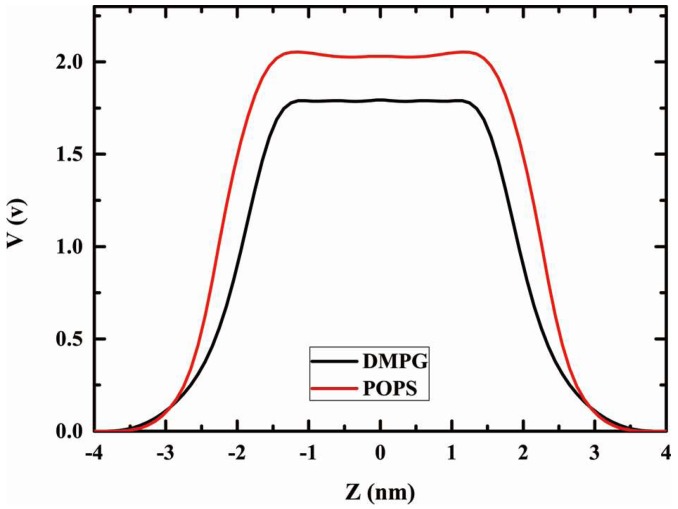
The electrostatic potential for membrane bilayer.

The induced dipole at each atomic site can be obtained from the simulations. Figure 9 shows the profile of electrostatic potential computed from the simulation. The potential difference [64] was calculated from Equation (8), and the values between water and membrane are 1.79 V for DMPG and 2.03 V for POPS. The potential barrier at the lipid-water interface was shifted from the center of bilayer, which derives from the strong ordering of water molecules around the phosphate group of the lipids. This is primarily due to differences in electrostatic representation. Induced polarization in the polarization in the interfacial region, is a particularly important property that cannot be captured by a force field with fixed partial charges.

## 3. Computational Details

**Figure 10:**
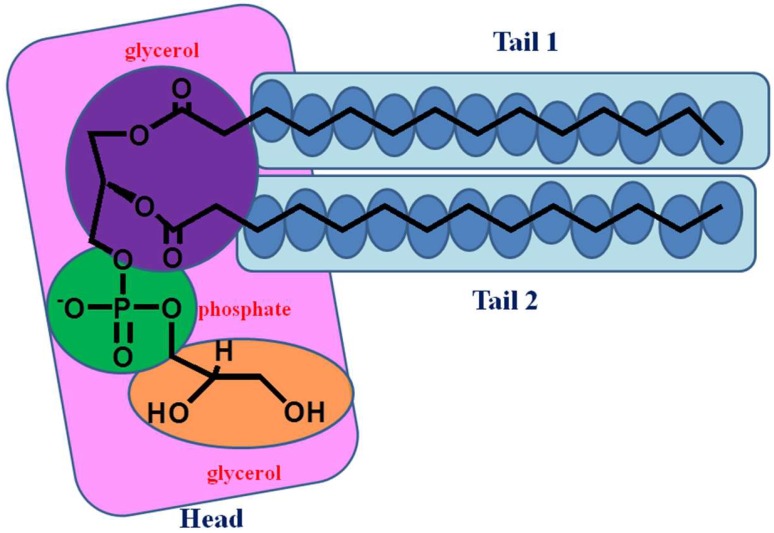
Illustration of small fragments used to construct the whole DMPG model. The hydrophilic headgroup (colored pink) was divided into three small functional fragments, which are two glycerol, and phosphate models, colored in purple, yellow and green, respectively. Every -CH_2_- group was the same small fragment in the two alkane tails, colored in blue. The whole two tails fragments are colored in cyan.

The lipid molecule contains charged hydrophilic headgroups, which interact with polar water, as well as hydrophobic tails, which meet with the hydrophobic tails of the complementary layer. The whole lipid molecule was first divided into several small fragments, and the terminated bonds were capped with -CH_3_ groupw (take the DMPG as an example shown in Figure 10).

The charged hydrophilic headgroup was divided into three small functional fragments, including two glycerol models (charge value 0), and phosphate group (charge value −1) for the PG headgroup (charge value −1), and glycerol (charge value 0), serine (charge value 0), and phosphate (charge value −1) models for PS headgroup (charge value −1). Each -CH_2_- group, except the ones bonded to double bond carbon, is considered as the same small fragment in the two-alkane tails. Double bond and adjacent -CH_2_- is also separated as a fragment. Gaussian 09 [65] and ORCA 3.0 [66] were used for ab initio calculations. Geometry optimization was performed at MP2/6-31G(*) level for these small fragments. Initial atomic multipoles for small fragments were derived at the MP2/6-311G** level of theory and basis set via Stone’s original DMA procedure [51] using the GDMA program. It arranges multipole sites at atomic centers and analytically assigns initial multipoles based on the electron density matrix. In GMDA, “Switch 0” and “Radius H 0.65” were set to access the original DMA procedure. Atomic polarizabilities were assigned based on the element type of each atom following the original AMOEBA procedure [67]. Polarization groups were partitioned between rotatable bonds to avoid dividing up chemical functional groups. In addition, a grid of electrostatic potentials around the molecular fragments was constructed from electron density calculations using MP2/6-311++G(2d,2p) basis set. Then, the initial multipole parameters of the small fragments were optimized against the electrostatic potential (ESP) of three different conformers simultaneously with a 0.5 kcal mol^−1^ electron^−2^ gradient convergence criteria. The optimization was carried out using the program “POTENTIAL” in TINKER 5.1 [67].

The parameters of three large fragments, including the headgroup and the two hydrophobic tails, were next derived from these small fragments. The atomic multipoles of these large fragments were directly derived from those of constituent small fragments and then optimized against MP2/6-311++G(2d,2p) ESP of the large fragments. Four different conformations of the headgroup and each of the hydrophobic tails were used in the ESP fitting. From Figure 10, it should be noted that the headgroup was extended to the C2 in the tails, and two cap groups -CH_3_. The atomic multipoles of the cap groups were set to “zero” during the headgroup ESP fitting. During the merging, the residual partial charge value is assigned to all heavy atoms in headgroup equally. Next, with the atomic charges fixed, the atomic dipole and quadrupole moments were optimized by fitting to the ESP of the four conformers of the whole headgroup with a 0.1 kcal mol^−1^ electron^−2^ gradient convergence criteria. Similar procedure was also applied to the parameterization of the two tails, which started from the C3 group in the tails, plus capping groups -CH_3_. Due to the fact that the dipole and quadrupole moments of tail atoms are quite small, the ESP optimization converged below 0.01 kcal mol^−1^ electron^−2^. The multipoles of headgroup, hydrophobic tails were then transferred to the whole lipid molecule without further optimization. The electrostatic potential around the molecule was calculated with permanent multipoles and induced-dipoles. In accordance with the solvation study conducted by Shi et al. [54], quadrupoles of hydroxyl groups were scaled by 60% after electrostatic potential fitting.

The initial valence and van der Waals parameters were transferred from the previous set derived from liquid simulations of small organic molecules containing similar functional groups [52,54]. The equilibrium values of bonds and angles were also modified according to QM optimized geometries. Torsional parameters about rotatable bonds were obtained by comparing the conformational energy profiles calculated from QM with the AMOEBA model after the electrostatics, vdW, and other valence parameters were derived. The dihedral angle was scanned by minimizing the torsions about the interest rotatable bond at 30° intervals with restraints. The 6th order Fourier series was then fit to the difference between the QM (MP2/6-31G**) and AMOEBA conformational energy without the specific torsion term (parameters set to 0) [68].

The molecular simulations of aqueous lipid membrane bilayer were performed using OpenMM for AMOEBA [19]. In each simulation, we used two PBC systems of lipid bilayers consisting of 72 lipid with water molecules, and each system was performed two separately simulation. The initial geometry for AMOEBA simulation was built from CHARMM-GUI membrane builder website [69]. To neutralize the anionic lipid bilayers, 72 sodium ions are added in the system. Molecular dynamics simulations using AMOEBA force field were performed at 303.5 K under periodic boundary conditions. The Particle-Mesh Ewald (PME) method [70] for long-ranged electrostatic calculations was employed. In all MD simulations, the time step was 2 fs with a direct-space vdW cutoff of 12 Å and Ewald cutoff of 7 Å. Mutual polarization was used to get the induced dipole moment in every integration step and the convergence threshold of the self-consistent induced dipole was set to 10^−5^ Dye. A Monte Carlo semi-isotropic barostat was used to maintain constant pressure. The tensionless simulation was performed, which the constant surface tension is set to 0 dyn/cm.

## 4. Conclusions

The polarizable force field following the same framework of the AMOEBA force field expanded to DMPG and POPS anionic lipids have been presented, based on the DMPC force field [36]. The mutual induction model was adopted, and the electrostatic parameters (atomic multipoles) were obtained from the MP2 calculations of fragments. The torsion parameters were obtained by comparing the conformational energy profile calculated from QM with the AMOEBA model. The AMOEBA model was subsequently validated through a series of dimer association energy calculations and liquid simulations. In this study, the interaction dimers include the the PG headgroup with water, and the PS headgroup with Na^+^. The gas-phase association energy of these dimers and conformational energy calculated by AMOEBA are in agreement with the QM results.

The final polarizable multipole anionic lipids force field was applied to the simulation of the systems of 72 DMPG and 72 POPS lipids with water molecules. The values of the area per lipid, volume per lipid, order parameters of tails, electron density profiles, lipid orientation and dynamics, and dipole potential were validated. The area per lipid produced by AMOEBA was closed to the experimental values. The electrostatic potential difference was calculated for bilayer, considering the whole monopole, dipole and quadrupole contributions. At the same time, we recognize a few limitations in the current study, such as a small number of the lipid molecules used in the simulation system and our relatively short simulation lengths, but the development of the two new anionic lipid molecules was made possible by molecular simulation on anionic lipids with advanced electrostatic descriptions.

## Figures and Tables

**Table 1:** Structural properties of bilayers calculated via molecular simulation using AMOEBA force field and comparison to limited experimental data.

Lipid System	Area Per Lipid A_L_(Å^2^)	Volume Per Lipid V_L_(Å^3^)	Peak Distance D_HH_ (Å)
DMPG			
72	60.05 ± 0.73	1021.10	33.9
Experiment	62.5 (ref. [58])		33.8 (ref. [58])
POPS			
72	60.93 ± 0.55	1130.94	38.7
Experiment			

## Supplementary Materials

The Supplementary Materials are available online.
