# Supplementary material for: A Polarizable Atomic Multipole-Based Force Field for Molecular Dynamics Simulations of Anionic Lipids
Source: Molecules. 2017 Dec 31;23(1):77. doi: 10.3390/molecules23010077 (PMC6017617; doi:10.3390/molecules23010077)
Supplement: Supplementary file 1 [file molecules-23-00077-s001.pdf]

Polarizable Atomic Multipole-Based Force Field for Molecular Dynamics Simulations of Anionic Lipids

Huiying Chu,[1†] Xiangda Peng,[1,2†] Yan Li,[1†] Yuebin Zhang,[1]Guohui Li[1]*

[1] Laboratory of Molecular Modeling and Design, State key Laboratory of Molecular Reaction Dynamics, Dalian Institute of Chemical Physics, Chinese Academy of Science, 457 Zhongshan Road. Dalian, Liaoning, 116023, China

[2] University of Chinese Academy of Sciences, Chinese Academy of Sciences, Beijing 100049, China

* Address correspondence to these authors at ghli@dicp.ac.cn

† All authors contributed equally to this work

Table S1 Comparison of torsional conformation energies predict by AMOEBA and QM (MP2/6-31G(d,p)) (kcal/mol) .

	R2	Intercept	Slope	Min/ Max	STD error	Average error
PS-AMOEBA	0.92	-0.16	0.88	0.00/8.78	7.12	1.19
PG-AMOEBA	0.87	-1.61	0.88	0.00/5.56	2.27	1.27

```
      ##############################
      ##                          ##
      ##  Force Field Definition  ##
      ##                          ##
      ##############################


forcefield              AMOEBA-DMPG

bond-cubic              -2.55
bond-quartic            3.793125
angle-cubic             -0.014
angle-quartic           0.000056
angle-pentic            -0.0000007
angle-sextic            0.000000022
torsionunit             0.5
vdwtype                 BUFFERED-14-7
opbendunit              0.02191418
radiusrule              CUBIC-MEAN
radiustype              R-MIN
radiussize              DIAMETER
epsilonrule             HHG
dielectric              1.0
polarization            MUTUAL
vdw-12-scale            0.0
vdw-13-scale            0.0
vdw-14-scale            1.0
vdw-15-scale            1.0
mpole-12-scale          0.0
mpole-13-scale          0.0
mpole-14-scale          0.4
mpole-15-scale          0.8
polar-12-scale          0.0
polar-13-scale          0.0
polar-14-scale          1.0
polar-15-scale          1.0
polar-14-intra          0.5
direct-11-scale         0.0
direct-12-scale         1.0
direct-13-scale         1.0
direct-14-scale         1.0
mutual-11-scale         1.0
mutual-12-scale         1.0
mutual-13-scale         1.0
mutual-14-scale         1.0

      ##############################
      ##                          ##
```

Atom Type Definitions
##
##############################

atom	601	601	C1	"DMPG glycero1 C1 "	6	12.011	4
atom	602	602	H1	"DMPG glycero1 H1 "	1	1.008	1
atom	603	603	OH1	"DMPG glycero1 OH1 "	8	15.999	2
atom	604	604	HO1	"DMPG glycero1 HO1 "	1	1.008	1
atom	605	605	C2	"DMPG glycero1 C2 "	6	12.011	4
atom	606	606	H2	"DMPG glycero1 H2 "	1	1.008	1
atom	607	603	OH2	"DMPG glycero1 OH2 "	8	15.999	2
atom	608	604	HO2	"DMPG glycero1 HO2 "	1	1.008	1
atom	609	630	C3	"DMPG glycero1 C3 "	6	12.011	4
atom	610	608	H3	"DMPG glycero1 H3 "	1	1.008	1
atom	611	609	P1	"DMPG phosphate P1 "	15	30.974	4
atom	612	610	O3	"DMPG phosphate O3 "	8	15.999	1
atom	613	611	O2	"DMPG phosphate O2 "	8	15.999	2
atom	614	611	O4	"DMPG phosphate O4 "	8	15.999	2
atom	615	607	C4	"DMPG glycerol2 C4 "	6	12.011	4
atom	616	608	H4	"DMPG glycerol2 H4 "	1	1.008	1
atom	617	612	C5	"DMPG glycerol2 C5 "	6	12.011	4
atom	618	613	H5	"DMPG glycerol2 H5 "	1	1.008	1
atom	619	614	OG1	"DMPG glycerol2 OG1 "	8	15.999	2
atom	620	615	C1b	"DMPG acyl chainb C1 "	6	12.011	3
atom	621	616	O1b	"DMPG sn-2 O1b "	8	15.999	1
atom	622	617	C2b	"DMPG acyl chainb C2 "	6	12.011	4
atom	623	618	H2b	"DMPG acyl chainb H2 "	1	1.008	1
atom	624	631	C6	"DMPG glycerol2 C6 "	6	12.011	4
atom	625	608	H6	"DMPG glycerol2 H6 "	1	1.008	1
atom	626	614	OG2	"DMPG glycerol2 OG2 "	8	15.999	2
atom	627	615	C1a	"DMPG acyl chaina C1 "	6	12.011	3
atom	628	616	O1a	"DMPG sn-2 O1a "	8	15.999	1
atom	629	617	C2a	"DMPG acyl chaina C2 "	6	12.011	4
atom	630	618	H2a	"DMPG acyl chaina H2 "	1	1.008	1
atom	631	619	C3t	"DMPG acyl chainab C3"	6	12.011	4
atom	632	620	H3t	"DMPG acyl chainab H3"	1	1.008	1
atom	633	619	C4t	"DMPG acyl chainab C4"	6	12.011	4
atom	634	620	H4t	"DMPG acyl chainab H4"	1	1.008	1
atom	635	619	C5t	"DMPG acyl chainab C5"	6	12.011	4
atom	636	620	H5t	"DMPG acyl chainab H5"	1	1.008	1
atom	637	619	C6t	"DMPG acyl chainab C6"	6	12.011	4
atom	638	620	H6t	"DMPG acyl chainab H6"	1	1.008	1
atom	639	619	C7t	"DMPG acyl chainab C7"	6	12.011	4
atom	640	620	H7t	"DMPG acyl chainab H7"	1	1.008	1
atom	641	619	C8t	"DMPG acyl chainab C8"	6	12.011	4
atom	642	620	H8t	"DMPG acyl chainab H8"	1	1.008	1
atom	643	619	C9t	"DMPG acyl chainab C9"	6	12.011	4
atom	644	620	H9t	"DMPG acyl chainab H9"	1	1.008	1
atom	645	619	C10	"DMPG acyl chainab C10"	6	12.011	4

atom	646	620	H10	"DMPG acyl chainab H10"	1	1.008	1
atom	647	619	C11	"DMPG acyl chainab C11"	6	12.011	4
atom	648	620	H11	"DMPG acyl chainab H11"	1	1.008	1
atom	649	619	C12	"DMPG acyl chainab C12"	6	12.011	4
atom	650	620	H12	"DMPG acyl chainab H12"	1	1.008	1
atom	651	619	C13	"DMPG acyl chainab C13"	6	12.011	4
atom	652	620	H13	"DMPG acyl chainab H13"	1	1.008	1
atom	653	621	C14	"DMPG acyl chainab C14"	6	12.011	4
atom	654	622	H14	"DMPG acyl chainab H14"	1	1.008	1

##################################
##
Van der Waals Parameters
##
##################################

vdw	601	3.8200	0.1010	
vdw	602	2.8700	0.0240	0.910
vdw	603	3.4050	0.1100	
vdw	604	2.6550	0.0135	0.910
vdw	605	3.6500	0.1010	
vdw	606	2.8700	0.0240	0.910
vdw	607	3.8200	0.1010	
vdw	608	2.9800	0.0240	0.940
vdw	609	4.4500	0.3900	
vdw	610	3.6300	0.1120	
vdw	611	3.4050	0.1100	
vdw	612	3.6500	0.1010	
vdw	613	2.9800	0.0240	0.940
vdw	614	3.4050	0.1100	
vdw	615	3.8200	0.1060	
vdw	616	3.3000	0.1120	
vdw	617	3.8200	0.1010	
vdw	618	2.9800	0.0240	0.940
vdw	619	3.8200	0.1010	
vdw	620	2.9800	0.0240	0.920
vdw	621	3.8200	0.1010	
vdw	622	2.9600	0.0240	0.920

####################################
##
Bond Stretching Parameters

##
##################################

bond	601	605	323.0000	1.5170
bond	601	603	465.1000	1.4024
bond	601	602	341.0000	1.0836
bond	603	604	615.9000	0.9509
bond	605	603	465.1000	1.4129
bond	605	606	341.0000	1.0840
bond	630	605	323.0000	1.5201
bond	630	611	465.1000	1.4011
bond	630	608	341.0000	1.0824
bond	607	612	323.0000	1.5169
bond	607	611	465.1000	1.3921
bond	607	614	465.1000	1.4234
bond	607	608	341.0000	1.0842
bond	609	611	450.0000	1.6479
bond	609	610	775.0000	1.4679
bond	612	614	465.1000	1.4284
bond	612	613	341.0000	1.0800
bond	615	614	465.1000	1.3199
bond	615	616	601.8000	1.1906
bond	617	615	345.3000	1.5107
bond	617	618	341.0000	1.0857
bond	619	617	323.0000	1.5233
bond	619	620	341.0000	1.0837
bond	631	612	323.0000	1.5151
bond	631	614	465.1000	1.4234
bond	631	608	341.0000	1.0792
bond	619	621	323.0000	1.5299
bond	621	622	341.0000	1.0861
bond	619	619	453.0000	1.5312
bond	619	620	341.0000	1.0868

#################################
##
Angle Bending Parameters
##
#################################

angle	605	601	603	88.0000	111.3169
angle	605	601	602	42.4400	109.3794
angle	603	601	602	60.9900	110.1884
angle	602	601	602	40.5700	108.1787
angle	601	603	604	64.9600	106.5531

angle	605	603	604	64.9600	106.7288
angle	630	605	601	48.2000	113.3604
angle	630	605	603	88.0000	109.6695
angle	630	605	606	42.4400	108.4718
angle	601	605	603	88.0000	105.8246
angle	601	605	606	42.4400	109.7398
angle	603	605	606	60.9900	109.7272
angle	605	630	611	88.0000	108.9330
angle	605	630	608	42.4400	109.6664
angle	611	630	608	60.9900	107.2293
angle	608	630	608	39.5700	108.8792
angle	612	631	614	88.0000	108.6276
angle	612	607	608	38.0000	110.9853
angle	614	607	608	60.9900	109.4841
angle	608	607	608	39.5700	108.5174
angle	612	607	611	88.0000	107.9292
angle	611	607	608	60.9900	110.5513
angle	611	609	611	65.5800	99.1337
angle	611	609	610	75.8600	108.2462
angle	610	609	610	89.8800	125.6037
angle	630	611	609	80.3000	119.8591
angle	607	611	609	80.3000	118.3962
angle	631	612	607	60.0000	113.6797
angle	631	612	614	88.0000	110.2555
angle	631	612	613	38.0000	107.9899
angle	614	612	613	60.9900	108.4937
angle	607	612	614	88.0000	107.1445
angle	607	612	613	38.0000	109.1704
angle	612	614	615	88.5000	119.5871
angle	607	614	609	80.3000	118.3962
angle	617	615	614	60.0000	111.0471
angle	617	615	616	60.0000	125.0470
angle	614	615	616	50.0000	123.9059
angle	631	614	615	88.5000	118.0991
angle	619	617	615	48.2000	113.1056
angle	619	617	618	38.0000	111.3844
angle	615	617	618	38.0000	107.3072
angle	618	617	618	45.5700	105.7941
angle	617	619	620	42.4400	111.1182
angle	620	619	620	39.5700	107.6064
angle	612	631	608	38.0000	110.9853
angle	614	631	608	60.9900	109.4841
angle	608	631	608	39.5700	108.5174
angle	619	619	619	48.2500	113.3100
angle	619	619	620	37.9900	109.4700
angle	620	619	621	37.9900	109.3700
angle	617	619	619	48.2000	112.2989
angle	622	621	622	39.5100	107.6500
angle	620	619	620	39.6600	106.2700

angle	619	619	621	48.2900	112.8700
angle	619	621	622	42.4200	111.2700
angle	619	619	623	48.2000	112.8390
angle	619	623	623	60.0000	125.4116
angle	619	623	624	32.0000	115.6966
angle	623	623	624	32.0000	118.8892
angle	620	619	623	38.0000	108.7346

################################
##
Stretch-Bend Parameters
##
################################

strbnd	605	601	603	18.7000	18.7000
strbnd	605	601	602	11.5000	11.5000
strbnd	603	601	602	-4.5000	38.0000
strbnd	605	603	604	-4.5000	38.0000
strbnd	601	603	604	-4.5000	38.0000
strbnd	630	605	601	38.0000	38.0000
strbnd	630	605	603	18.7000	18.7000
strbnd	630	605	606	-4.5000	38.0000
strbnd	601	605	603	18.7000	18.7000
strbnd	601	605	606	-4.5000	38.0000
strbnd	607	605	606	-4.5000	38.0000
strbnd	612	607	614	18.7000	18.7000
strbnd	612	607	608	11.5000	18.7000
strbnd	614	607	608	-4.5000	38.0000
strbnd	612	631	614	18.7000	18.7000
strbnd	612	631	608	11.5000	18.7000
strbnd	614	631	608	-4.5000	38.0000
strbnd	612	607	611	18.7000	18.7000
strbnd	611	607	608	-4.5000	38.0000
strbnd	611	609	611	14.4000	14.4000
strbnd	611	609	610	14.4000	14.4000
strbnd	607	611	609	38.0000	38.0000
strbnd	607	612	607	18.7000	18.7000
strbnd	607	612	614	18.7000	18.7000
strbnd	607	612	613	11.5000	18.7000
strbnd	614	612	613	-4.5000	38.0000
strbnd	612	614	620	38.0000	38.0000
strbnd	607	614	615	38.0000	38.0000
strbnd	631	614	615	38.0000	38.0000
strbnd	617	615	614	18.7000	18.7000
strbnd	619	617	615	18.7000	18.7000
strbnd	619	617	618	11.5000	18.7000

```
strbnd    615    617    618    11.5000    18.7000
strbnd    617    620    614    18.7000    18.7000
strbnd    617    621    620    11.5000    11.5000
strbnd    605    630    611    18.7000    18.7000
strbnd    605    630    608    11.5000    11.5000
strbnd    611    630    608    -4.5000    38.0000
strbnd    615    620    619    38.0000    38.0000
strbnd    609    620    619    38.0000    38.0000
strbnd    617    619    620    11.5000    11.5000
strbnd    619    619    619    18.7000    18.7000
strbnd    619    619    620    11.5000    18.7000
strbnd    621    619    620    11.5000    18.7000
strbnd    623    619    624    11.5000    11.5000
strbnd    617    619    619    18.7000    18.7000
strbnd    619    617    618    11.5000    18.7000
strbnd    619    619    623    18.7000    18.7000
strbnd    620    619    623    11.5000    18.7000
strbnd    619    621    622    11.5000    11.5000
strbnd    617    619    620    11.5000    18.7000
strbnd    619    623    624    11.5000    18.7000
strbnd    623    619    623    18.7000    18.7000
strbnd    623    619    624    11.5000    18.7000
strbnd    624    623    623    11.5000    18.7000


      ####################################
      ##                                ##
      ##  Out-of-Plane Bend Parameters  ##
      ##                                ##
      ####################################


opbend    617    615    0    0    0.2002
opbend    614    615    0    0    0.2002
opbend    616    615    0    0    0.6500

      ############################
      ##                        ##
      ##  Torsional Parameters  ##
      ##                        ##
      ############################


torsion    603    601    605    630    -0.253 0.0 1    0.007 180.0 2    1.585 0.0 3
torsion    603    601    605    603     0.567 0.0 1   -0.739 180.0 2    1.585 0.0 3
```

torsion	603	601	605	606	0.000 0.0 1	0.000 180.0 2	0.000 0.0 3
torsion	602	601	605	630	0.000 0.0 1	0.000 180.0 2	0.000 0.0 3
torsion	602	601	605	603	0.000 0.0 1	0.000 180.0 2	0.000 0.0 3
torsion	602	601	605	606	0.000 0.0 1	0.000 180.0 2	0.000 0.0 3
torsion	605	601	603	604	-1.447 0.0 1	0.531 180.0 2	0.317 0.0 3
torsion	602	601	603	604	0.000 0.0 1	0.000 180.0 2	0.274 0.0 3
torsion	630	605	603	604	-1.372 0.0 1	0.232 180.0 2	0.400 0.0 3
torsion	601	605	603	604	-1.372 0.0 1	0.232 180.0 2	0.400 0.0 3
torsion	606	605	603	604	0.000 0.0 1	0.000 180.0 2	0.266 0.0 3
torsion	605	630	611	609	-5.690 0.0 1	-0.340 180.0 2	-1.004 0.0 3
torsion	608	607	612	631	0.000 0.0 1	0.000 180.0 2	0.000 0.0 3
torsion	608	607	612	614	0.000 0.0 1	0.000 180.0 2	0.000 0.0 3
torsion	608	631	612	607	0.000 0.0 1	0.000 180.0 2	0.000 0.0 3
torsion	608	631	612	614	0.000 0.0 1	0.000 180.0 2	0.000 0.0 3
torsion	608	630	612	607	0.000 0.0 1	0.000 180.0 2	0.000 0.0 3
torsion	608	630	605	601	0.000 0.0 1	0.000 180.0 2	0.000 0.0 3
torsion	608	630	605	603	0.000 0.0 1	0.000 180.0 2	0.000 0.0 3
torsion	608	630	605	606	0.000 0.0 1	0.000 180.0 2	0.000 0.0 3
torsion	608	607	611	609	0.000 0.0 1	0.000 180.0 2	0.000 0.0 3
torsion	608	631	612	613	0.000 0.0 1	0.000 180.0 2	0.000 0.0 3
torsion	608	607	612	613	0.000 0.0 1	0.000 180.0 2	0.000 0.0 3
torsion	608	631	614	615	0.000 0.0 1	0.000 180.0 2	0.000 0.0 3
torsion	608	630	611	609	0.000 0.0 1	0.000 180.0 2	0.000 0.0 3
torsion	611	630	605	601	-10.510 0.0 1	-1.120 180.0 2	-0.145 0.0 3
torsion	611	630	605	603	-10.337 0.0 1	-2.220 180.0 2	1.661 0.0 3
torsion	611	630	605	606	0.000 0.0 1	0.000 180.0 2	0.000 0.0 3
torsion	611	607	612	631	2.061 0.0 1	-0.273 180.0 2	0.000 0.0 3
torsion	611	607	612	614	2.473 0.0 1	-0.641 180.0 2	0.000 0.0 3
torsion	611	607	612	613	0.000 0.0 1	0.000 180.0 2	0.000 0.0 3
torsion	612	631	614	615	0.950 0.0 1	-0.615 180.0 2	-0.900 0.0 3
torsion	612	607	611	609	-3.099 0.0 1	-1.371 180.0 2	-0.640 0.0 3
torsion	614	631	612	607	0.756 0.0 1	-0.023 180.0 2	0.788 0.0 3
torsion	614	631	612	614	0.220 0.0 1	-0.592 180.0 2	0.788 0.0 3
torsion	614	631	612	613	0.000 0.0 1	0.000 180.0 2	0.000 0.0 3
torsion	611	609	611	630	3.996 0.0 1	-2.322 180.0 2	0.547 0.0 3
torsion	610	609	611	630	0.000 0.0 1	0.000 180.0 2	0.000 0.0 3
torsion	611	609	611	607	0.000 0.0 1	0.000 180.0 2	4.835 0.0 3
torsion	610	609	611	607	0.000 0.0 1	0.000 180.0 2	-2.217 0.0 3
torsion	631	612	614	615	5.257 0.0 1	-0.267 180.0 2	-3.761 0.0 3
torsion	607	612	614	615	3.413 0.0 1	-1.059 180.0 2	2.755 0.0 3
torsion	613	612	614	615	0.000 0.0 1	0.000 180.0 2	0.000 0.0 3
torsion	617	615	614	631	0.000 0.0 1	5.965 180.0 2	0.000 0.0 3
torsion	616	615	614	631	0.000 0.0 1	5.965 180.0 2	0.000 0.0 3
torsion	617	615	614	612	0.000 0.0 1	5.294 180.0 2	0.000 0.0 3
torsion	616	615	614	612	0.000 0.0 1	5.294 180.0 2	0.000 0.0 3
torsion	619	617	615	614	1.231 0.0 1	0.000 180.0 2	0.000 0.0 3
torsion	619	617	615	616	0.000 0.0 1	-0.039 180.0 2	0.000 0.0 3
torsion	618	617	615	614	0.000 0.0 1	0.000 180.0 2	0.000 0.0 3
torsion	618	617	615	616	0.000 0.0 1	0.000 180.0 2	0.000 0.0 3

```
torsion   615  617  619  620    0.000 0.0 1    0.000 180.0 2    0.000 0.0 3
torsion   615  617  619  619    1.532 0.0 1   -1.981 180.0 2    1.641 0.0 3
torsion   618  617  619  619    0.000 0.0 1    0.000 180.0 2    0.000 0.0 3
torsion   615  617  619  620    0.000 0.0 1    0.000 180.0 2    0.000 0.0 3
torsion   618  617  619  620    0.000 0.0 1    0.000 180.0 2    0.000 0.0 3
torsion   614  615  617  619    0.000 0.0 1   -0.039 180.0 2    0.000 0.0 3
torsion   622  621  619  620    0.000 0.0 1    0.000 180.0 2    0.299 0.0 3
torsion   620  619  623  623    0.000 0.0 1    0.000 180.0 2    0.000 0.0 3
torsion   620  619  623  624    0.000 0.0 1    0.000 180.0 2    0.000 0.0 3
torsion   617  619  619  619    2.818 0.0 1   -0.410 180.0 2    2.472 0.0 3
torsion   619  619  619  620    0.000 0.0 1    0.000 180.0 2    0.000 0.0 3
torsion   617  619  619  620    0.000 0.0 1    0.000 180.0 2    0.000 0.0 3
torsion   620  619  619  620    0.000 0.0 1    0.000 180.0 2    0.299 0.0 3
torsion   619  619  619  619    0.568 0.0 1   -0.375 180.0 2    2.302 0.0 3
torsion   619  623  623  619    0.000 0.0 1    8.000 180.0 2    0.000 0.0 3
torsion   619  619  623  623    2.775 0.0 1   -0.695 180.0 2   -1.271 0.0 3
torsion   619  623  623  624    0.000 0.0 1    6.100 180.0 2    0.000 0.0 3
torsion   624  623  623  624    0.000 0.0 1    4.070 180.0 2    0.000 0.0 3
torsion   619  619  623  624    0.000 0.0 1    0.000 180.0 2    0.000 0.0 3
torsion   620  619  619  623    0.000 0.0 1    0.000 180.0 2    0.000 0.0 3
torsion   619  619  619  623    0.649 0.0 1    0.419 180.0 2    2.472 0.0 3
torsion   619  619  619  621    0.717 0.0 1   -0.133 180.0 2    1.123 0.0 3
torsion   620  619  619  621    0.000 0.0 1    0.000 180.0 2    0.341 0.0 3
torsion   622  621  619  619    0.000 0.0 1    0.000 180.0 2    0.341 0.0 3


      #####################################
      ##                                 ##
      ##  Atomic Multipole Parameters    ##
      ##                                 ##
      #####################################


multipole   601  605  603               0.14321
                                          0.32240    0.00000    0.09462
                                          0.23810
                                          0.00000   -0.37250
                                         -0.36896    0.00000    0.13440
multipole   602  601  605               0.00784
                                         -0.02351    0.00000   -0.15975
                                          0.07822
                                          0.00000    0.11571
                                         -0.04421    0.00000   -0.19393
multipole   603  601  604              -0.39070
                                          0.18127    0.00000    0.29036
                                          0.30351
                                          0.00000   -0.95004
                                         -0.71221    0.00000    0.64653
```

multipole 604 603 601 0.22528
-0.03855 0.00000 -0.03655
-0.07847
0.00000 -0.11637
-0.23815 0.00000 0.19484
multipole 605 609 601 0.06538
0.01518 0.00000 0.02809
0.00730
0.00000 -0.00349
0.16576 0.00000 -0.00381
multipole 606 605 609 0.03496
0.03145 0.00000 -0.00998
-0.04100
0.00000 0.11430
0.05911 0.00000 -0.07330
multipole 607 605 608 -0.34019
0.17501 0.00000 0.32600
0.28616
0.00000 -0.85046
-0.52577 0.00000 0.56430
multipole 608 607 605 0.21690
-0.08046 0.00000 -0.03557
-0.08494
0.00000 -0.02354
-0.08847 0.00000 0.10848
multipole 609 605 613 0.12717
0.39300 0.00000 0.04872
0.42188
0.00000 -0.43739
-0.47076 0.00000 0.01551
multipole 610 609 605 0.02153
-0.01988 0.00000 -0.04884
-0.04385
0.00000 -0.00397
0.01708 0.00000 0.04782
multipole 611 612 -612 1.75436
0.00000 0.00000 -0.02762
0.11169
0.00000 0.13248
0.00000 0.00000 -0.24417
multipole 612 611 612 -0.94669
0.00000 0.00000 -0.04937
-0.20908
0.00000 -0.25330
0.00000 0.00000 0.46238
multipole 613 609 611 -0.53817
0.10997 0.00000 0.35554
-0.36722
0.00000 -0.82584

-0.21989 0.00000 1.19306
multipole 614 615 611 -0.53817
-0.03340 0.00000 0.43339
-0.44704
0.00000 -0.62023
-0.16194 0.00000 1.06727
multipole 615 617 614 0.12717
0.36195 0.00000 0.06164
0.26076
0.00000 -0.49801
-0.09726 0.00000 0.23725
multipole 616 615 617 0.02153
0.00104 0.00000 -0.06459
-0.04854
0.00000 0.01303
-0.09648 0.00000 0.03551
multipole 617 624 615 0.02657
0.04187 0.00000 0.22345
0.15487
0.00000 -0.02748
-0.17760 0.00000 -0.12739
multipole 618 617 624 0.08536
0.01608 0.00000 0.01881
-0.09901
0.00000 0.07857
0.03773 0.00000 0.02044
multipole 619 617 620 -0.29195
0.09319 0.00000 0.43233
-0.15745
0.00000 -0.33908
-0.17964 0.00000 0.49653
multipole 620 622 619 0.78665
0.02327 0.00000 -0.21575
0.35613
0.00000 -0.07671
-0.18339 0.00000 -0.27942
multipole 621 620 622 -0.62869
0.07600 0.00000 -0.00665
-0.50354
0.00000 0.18185
0.20943 0.00000 0.32169
multipole 622 631 620 -0.15650
0.13286 0.00000 0.20347
0.25455
0.00000 -0.51114
-0.12848 0.00000 0.25659
multipole 623 622 631 0.08635
0.04592 0.00000 -0.08127
0.02657

				0.00000	-0.01148	
				0.13451	0.00000	-0.01509
multipole	624	617	626	0.08356		
				0.36212	0.00000	0.14124
				0.53656		
				0.00000	-0.37503	
				-0.48187	0.00000	-0.16153
multipole	625	624	617	0.03662		
				-0.07375	0.00000	-0.09505
				0.01557		
				0.00000	0.06592	
				-0.06368	0.00000	-0.08149
multipole	626	624	627	-0.36542		
				0.10786	0.00000	0.28569
				-0.27399		
				0.00000	-0.62678	
				-0.36403	0.00000	0.90077
multipole	627	629	626	0.96543		
				-0.15609	0.00000	0.00907
				0.47252		
				0.00000	-0.19153	
				-0.32984	0.00000	-0.28099
multipole	628	627	629	-0.67403		
				-0.08121	0.00000	-0.08123
				-0.31575		
				0.00000	0.19743	
				0.04400	0.00000	0.11832
multipole	629	631	627	-0.33818		
				-0.09050	0.00000	0.28717
				-0.15732		
				0.00000	-0.14994	
				0.24675	0.00000	0.30726
multipole	630	629	631	0.08282		
				0.05263	0.00000	-0.10003
				0.04780		
				0.00000	0.07340	
				0.12940	0.00000	-0.12120
multipole	631	633	629	-0.12028		
				0.00000	0.00000	0.08920
				-0.45259		
				0.00000	0.22212	
				0.00000	0.00000	0.23047
multipole	631	633	622	-0.12028		
				0.00000	0.00000	0.08920
				-0.45259		
				0.00000	0.22212	
				0.00000	0.00000	0.23047
multipole	632	631	633	0.06014		
				-0.02503	0.00000	0.07942

-0.13076
0.00000 -0.05780
-0.05505 0.00000 0.18856
multipole 633 635 631 -0.12028
0.00000 0.00000 0.33839
-0.68115
0.00000 0.10340
0.00000 0.00000 0.57775
multipole 634 633 635 0.06014
0.00661 0.00000 0.11930
-0.16797
0.00000 -0.07505
0.12169 0.00000 0.24302
multipole 635 633 637 -0.12028
0.00000 0.00000 0.14448
-0.28128
0.00000 -0.00441
0.00000 0.00000 0.28569
multipole 636 635 633 0.06014
0.01615 0.00000 0.22955
-0.21643
0.00000 -0.20428
0.02788 0.00000 0.42071
multipole 637 635 639 -0.12028
0.00000 0.00000 -0.01354
-0.04975
0.00000 0.10300
0.00000 0.00000 -0.05325
multipole 638 637 635 0.06014
0.04578 0.00000 0.17404
-0.16537
0.00000 -0.18492
0.07403 0.00000 0.35029
multipole 639 637 641 -0.12028
0.00000 0.00000 -0.05085
-0.30712
0.00000 0.15148
0.00000 0.00000 0.15564
multipole 640 639 637 0.06014
0.08671 0.00000 0.09820
-0.08927
0.00000 -0.11428
0.11609 0.00000 0.20355
multipole 641 639 -643 -0.12028
0.00000 0.00000 -0.02523
0.11672
0.00000 0.26467
0.00000 0.00000 -0.38139
multipole 642 641 639 0.06014

				-0.00822	0.00000	0.09256
				-0.12801		
				0.00000	-0.10497	
				-0.03016	0.00000	0.23298
multipole	643	645	641	-0.12028		
				0.00000	0.00000	0.04373
				-0.11409		
				0.00000	0.25407	
				0.00000	0.00000	-0.13998
multipole	644	643	645	0.06014		
				0.03147	0.00000	0.10815
				-0.04700		
				0.00000	-0.16314	
				0.06910	0.00000	0.21014
multipole	645	647	643	-0.12028		
				0.00000	0.00000	0.04534
				0.03346		
				0.00000	0.20973	
				0.00000	0.00000	-0.24319
multipole	646	645	647	0.06014		
				0.02363	0.00000	0.17973
				-0.11928		
				0.00000	-0.23560	
				0.06988	0.00000	0.35488
multipole	647	649	645	-0.12028		
				0.00000	0.00000	0.19090
				-0.13792		
				0.00000	0.13912	
				0.00000	0.00000	-0.00120
multipole	648	647	649	0.06014		
				-0.00563	0.00000	0.24246
				-0.19465		
				0.00000	-0.25447	
				0.03243	0.00000	0.44912
multipole	649	647	651	-0.12028		
				0.00000	0.00000	0.17802
				-0.14130		
				0.00000	-0.03152	
				0.00000	0.00000	0.17282
multipole	650	649	647	0.06014		
				0.01343	0.00000	0.22793
				-0.19168		
				0.00000	-0.23346	
				0.05530	0.00000	0.42514
multipole	651	649	653	-0.12028		
				0.00000	0.00000	-0.04785
				-0.24581		
				0.00000	-0.05659	
				0.00000	0.00000	0.30240

```
multipole   652  651  649              0.06014
                                         0.10189    0.00000    0.12283
                                        -0.11650
                                         0.00000   -0.13166
                                         0.14624    0.00000    0.24816
multipole   653  651  654             -0.16638
                                         0.00000    0.00000    0.13817
                                         0.23776
                                         0.00000    0.30333
                                         0.00000    0.00000   -0.54109
multipole   654  653  651              0.05546
                                        -0.02203    0.00000    0.07426
                                        -0.02038
                                         0.00000   -0.17435
                                        -0.01461    0.00000    0.19473


      ##########################################
      ##                                      ##
      ##  Dipole Polarizability Parameters  ##
      ##                                      ##
      ##########################################


polarize   601    1.334     0.390    602 603
polarize   602    0.496     0.390    601
polarize   603    0.837     0.390    601 604
polarize   604    0.496     0.390    603
polarize   605    1.334     0.390    606 607
polarize   606    0.496     0.390    605
polarize   607    0.837     0.390    605 608
polarize   608    0.496     0.390    607
polarize   609    1.334     0.390    610 613
polarize   610    0.496     0.390    609
polarize   611    1.828     0.390    612 613 614
polarize   612    0.837     0.390    611
polarize   613    0.837     0.390    609 611
polarize   614    0.837     0.390    611 615
polarize   615    1.334     0.390    614 616
polarize   616    0.496     0.390    615
polarize   617    1.334     0.390    618 619
polarize   618    0.496     0.390    617
polarize   619    0.837     0.390    617 620
polarize   620    1.334     0.390    619 621 622
polarize   621    0.837     0.390    620
polarize   622    1.334     0.390    620 623
polarize   623    0.496     0.390    622
polarize   624    1.334     0.390    608 626
polarize   625    0.496     0.390    624
```

polarize	626	0.837	0.390	624 627
polarize	627	1.334	0.390	626 628 629
polarize	628	0.837	0.390	627
polarize	629	1.334	0.390	627 630
polarize	630	0.496	0.390	629
polarize	631	1.334	0.390	633 632
polarize	632	0.496	0.390	631
polarize	633	1.334	0.390	631 634
polarize	634	0.496	0.390	633
polarize	635	1.334	0.390	637 636
polarize	636	0.496	0.390	635
polarize	637	1.334	0.390	635 638
polarize	638	0.496	0.390	637
polarize	639	1.334	0.390	640 641
polarize	640	0.496	0.390	639
polarize	641	1.334	0.390	642 639
polarize	642	0.496	0.390	641
polarize	643	1.334	0.390	645 644
polarize	644	0.496	0.390	643
polarize	645	1.334	0.390	643 646
polarize	646	0.496	0.390	645
polarize	647	1.334	0.390	648
polarize	648	0.496	0.390	647
polarize	649	1.334	0.390	650 651
polarize	650	0.496	0.390	649
polarize	651	1.334	0.390	649 652
polarize	652	0.496	0.390	651
polarize	653	1.334	0.390	654
polarize	654	0.496	0.390	653

```
###############################
##                           ##
##   Force Field Definition  ##
##                           ##
###############################


forcefield              AMOEBA-POPS

bond-cubic              -2.55
bond-quartic            3.793125
angle-cubic             -0.014
angle-quartic           0.000056
angle-pentic            -0.0000007
angle-sextic            0.000000022
torsionunit             0.5
vdwtype                 BUFFERED-14-7
opbendunit              0.02191418
radiusrule              CUBIC-MEAN
radiustype              R-MIN
radiussize              DIAMETER
epsilonrule             HHG
dielectric              1.0
polarization            MUTUAL
vdw-12-scale            0.0
vdw-13-scale            0.0
vdw-14-scale            1.0
vdw-15-scale            1.0
mpole-12-scale          0.0
mpole-13-scale          0.0
mpole-14-scale          0.4
mpole-15-scale          0.8
polar-12-scale          0.0
polar-13-scale          0.0
polar-14-scale          1.0
polar-15-scale          1.0
polar-14-intra          0.5
direct-11-scale         0.0
direct-12-scale         1.0
direct-13-scale         1.0
direct-14-scale         1.0
mutual-11-scale         1.0
mutual-12-scale         1.0
mutual-13-scale         1.0
mutual-14-scale         1.0

##############################
##                          ##
```

Atom Type Definitions
##
##############################

atom	801	801	N	"POPS serine N+ "	7	14.007	4
atom	802	802	H1	"POPS serine HN "	1	1.008	1
atom	803	803	CA	"POPS serine CA "	6	12.011	4
atom	804	804	HA	"POPS serine HA "	1	1.008	1
atom	805	805	CO	"POPS serine COO- "	6	12.011	3
atom	806	806	OC	"POPS serine O- "	8	15.999	1
atom	807	807	CB	"POPS serine CB "	6	12.011	4
atom	808	808	HB	"POPS serine HB "	1	1.008	1
atom	809	809	P1	"POPS phosphate P1 "	15	30.974	4
atom	810	810	O3	"POPS phosphate O3 "	8	15.999	1
atom	811	811	O2	"POPS phosphate O2 "	8	15.999	2
atom	812	811	O4	"POPS phosphate O4 "	8	15.999	2
atom	813	812	C1	"POPS glycero1 C1 "	6	12.011	4
atom	814	813	H1	"POPS glycero1 H1 "	1	1.008	1
atom	815	814	C2	"POPS glycerol C2 "	6	12.011	4
atom	816	815	H2	"POPS glycerol H2 "	1	1.008	1
atom	817	816	OG1	"POPS glycerol OG1 "	8	15.999	2
atom	818	817	C1B	"POPS acyl chainb C1 "	6	12.011	3
atom	819	818	O1B	"POPS sn-2 O1b "	8	15.999	1
atom	820	819	C2B	"POPS acyl chainb C2 "	6	12.011	4
atom	821	820	H2B	"POPS acyl chainb H2 "	1	1.008	1
atom	822	821	C3	"DLPG glycerol C3 "	6	12.011	4
atom	823	822	H3	"DLPG glycerol H3 "	1	1.008	1
atom	824	816	OG2	"POPS glycerol OG2 "	8	15.999	2
atom	825	817	C1A	"POPS acyl chaina C1 "	6	12.011	3
atom	826	818	O1A	"POPS sn-2 O1a "	8	15.999	1
atom	827	819	C2A	"POPS acyl chaina C2 "	6	12.011	4
atom	828	820	H2A	"POPS acyl chaina H2 "	1	1.008	1
atom	829	823	C3T	"POPS acyl chainab C3"	6	12.011	4
atom	830	824	H3T	"POPS acyl chainab H3"	1	1.008	1
atom	831	823	C4t	"POPS acyl chainab C4"	6	12.011	4
atom	832	824	H4t	"POPS acyl chainab H4"	1	1.008	1
atom	833	823	C5t	"POPS acyl chainab C5"	6	12.011	4
atom	834	824	H5t	"POPS acyl chainab H5"	1	1.008	1
atom	835	823	C6t	"POPS acyl chainab C6"	6	12.011	4
atom	836	824	H6t	"POPS acyl chainab H6"	1	1.008	1
atom	837	823	C7t	"POPS acyl chainab C7"	6	12.011	4
atom	838	824	H7t	"POPS acyl chainab H7"	1	1.008	1
atom	839	823	C8t	"POPS acyl chainab C8"	6	12.011	4
atom	840	824	H8t	"POPS acyl chainab H8"	1	1.008	1
atom	841	823	C9t	"POPS acyl chainab C9"	6	12.011	4
atom	842	824	H9t	"POPS acyl chainab H9"	1	1.008	1
atom	843	823	C10	"POPS acyl chainab C10"	6	12.011	4
atom	844	824	H10	"POPS acyl chainab H10"	1	1.008	1
atom	845	823	C11	"POPS acyl chainab C11"	6	12.011	4

atom	846	824	H11	"POPS acyl chainab H11"	1	1.008	1
atom	847	823	C12	"POPS acyl chainab C12"	6	12.011	4
atom	848	824	H12	"POPS acyl chainab H12"	1	1.008	1
atom	849	823	C13	"POPS acyl chainab C13"	6	12.011	4
atom	850	824	H13	"POPS acyl chainab H13"	1	1.008	1
atom	851	823	C14	"POPS acyl chainab C14"	6	12.011	4
atom	852	824	H14	"POPS acyl chainab H14"	1	1.008	1
atom	853	823	C15	"POPS acyl chainab C15"	6	12.011	4
atom	854	824	H15	"POPS acyl chainab H15"	1	1.008	1
atom	855	825	C16	"POPS acyl chainab C16"	6	12.011	4
atom	856	826	H16	"POPS acyl chainab H16"	1	1.008	1
atom	857	823	C3b	"POPS acyl chainb C3"	6	12.011	4
atom	858	824	H3b	"POPS acyl chainb H3"	1	1.008	1
atom	859	823	C4b	"POPS acyl chainb C4"	6	12.011	4
atom	860	824	H4b	"POPS acyl chainb H4"	1	1.008	1
atom	861	823	C5b	"POPS acyl chainb C5"	6	12.011	4
atom	862	824	H5b	"POPS acyl chainb H5"	1	1.008	1
atom	863	823	C6b	"POPS acyl chainb C6"	6	12.011	4
atom	864	824	H6b	"POPS acyl chainb H6"	1	1.008	1
atom	865	823	C7b	"POPS acyl chainb C7"	6	12.011	4
atom	866	824	H7b	"POPS acyl chainb H7"	1	1.008	1
atom	867	823	C8b	"POPS acyl chainb C8"	6	12.011	4
atom	868	824	H8b	"POPS acyl chainb H8"	1	1.008	1
atom	869	827	C9b	"POPS acyl chainb C9"	6	12.011	3
atom	870	828	H9b	"POPS acyl chainb H9"	1	1.008	1
atom	871	827	C10	"POPS acyl chainb C10"	6	12.011	3
atom	872	828	H10	"POPS acyl chainb H10"	1	1.008	1
atom	873	823	C11	"POPS acyl chainb C11"	6	12.011	4
atom	874	824	H11	"POPS acyl chainb H11"	1	1.008	1
atom	875	823	C12	"POPS acyl chainb C12"	6	12.011	4
atom	876	824	H12	"POPS acyl chainb H12"	1	1.008	1
atom	877	823	C13	"POPS acyl chainb C13"	6	12.011	4
atom	878	824	H13	"POPS acyl chainb H13"	1	1.008	1
atom	879	823	C14	"POPS acyl chainb C14"	6	12.011	4
atom	880	824	H14	"POPS acyl chainb H14"	1	1.008	1
atom	881	823	C15	"POPS acyl chainb C15"	6	12.011	4
atom	882	824	H15	"POPS acyl chainb H15"	1	1.008	1
atom	883	823	C16	"POPS acyl chainb C16"	6	12.011	4
atom	884	824	H16	"POPS acyl chainb H16"	1	1.008	1
atom	885	823	C17	"POPS acyl chainb C17"	6	12.011	4
atom	886	824	H17	"POPS acyl chainb H17"	1	1.008	1
atom	887	825	C18	"POPS acyl chainb C18"	6	12.011	4
atom	888	826	H18	"POPS acyl chainb H18"	1	1.008	1

```
################################
##                            ##
##   Van der Waals Parameters ##
##                            ##
################################

vdw     801   3.7100   0.1050
vdw     802   2.4800   0.0115 0.900
vdw     803   3.6500   0.1010
vdw     804   2.9800   0.0240 0.940
vdw     805   3.8200   0.1060
vdw     806   3.4500   0.1120
vdw     807   3.8200   0.1010
vdw     808   2.9800   0.0240 0.940
vdw     809   4.4500   0.3900
vdw     810   3.6300   0.1120
vdw     811   3.4050   0.1100
vdw     812   3.8200   0.1010
vdw     813   2.9800   0.0240 0.940
vdw     814   3.6500   0.1010
vdw     815   2.9800   0.0240 0.940
vdw     816   3.4050   0.1100
vdw     817   3.8200   0.1060
vdw     818   3.3000   0.1120
vdw     819   3.8200   0.1010
vdw     820   2.9800   0.0240 0.940
vdw     821   3.8200   0.1010
vdw     822   2.9800   0.0240 0.940
vdw     823   3.8200   0.1010
vdw     824   2.9600   0.0240 0.920
vdw     825   3.8200   0.1010
vdw     825   2.9600   0.0240 0.920
vdw     827   3.6500   0.1010
vdw     828   2.5830   0.0260 0.920



##################################
##                              ##
##   Bond Stretching Parameters ##
##                              ##
##################################

bond      801   803          381.3     1.4911
bond      801   802          461.9     1.0133
bond      803   805          323.0     1.5570
bond      803   804          341.0     1.0811
```

bond	805	806	601.8	1.2196
bond	807	803	323.0	1.5251
bond	807	811	465.1	1.4088
bond	807	808	341.0	1.0821
bond	809	811	450.0	1.6343
bond	809	810	775.0	1.4860
bond	812	811	465.1	1.4016
bond	812	813	341.0	1.0808
bond	814	812	323.0	1.5197
bond	814	821	323.0	1.5141
bond	814	816	465.1	1.4302
bond	814	815	341.0	1.0779
bond	817	816	465.1	1.3201
bond	817	818	601.8	1.1909
bond	819	823	323.0	1.5233
bond	819	817	345.3	1.5113
bond	819	820	341.0	1.0858
bond	821	816	465.1	1.4270
bond	821	822	341.0	1.0783
bond	819	823	323.0000	1.5231
bond	823	824	341.0000	1.0832
bond	823	825	323.0000	1.5299
bond	823	823	453.0000	1.5247
bond	825	826	341.0000	1.0861
bond	823	827	323.0000	1.5053
bond	827	827	680.0000	1.3201
bond	827	828	341.0000	1.0810

################################
##
Angle Bending Parameters
##
################################

angle	803	801	802	35.0000	110.9636
angle	802	801	802	34.5000	107.7790
angle	807	803	801	80.0000	109.9757
angle	807	803	805	60.0000	114.3450
angle	807	803	804	38.0000	110.0352
angle	801	803	805	80.0000	107.7150
angle	801	803	804	50.6000	105.0679
angle	805	803	804	38.0000	109.2687
angle	803	805	806	60.0000	113.4509
angle	806	805	806	57.6000	131.7978
angle	803	807	811	88.0000	113.6748
angle	803	807	808	38.0000	109.6972
angle	811	807	808	60.9900	105.8027
angle	808	807	808	39.5700	107.7343

angle	811	809	811	65.5800	100.9105
angle	811	809	810	75.8600	107.4376
angle	810	809	810	89.8800	123.0639
angle	807	811	809	80.3000	125.5126
angle	812	811	809	80.3000	119.2241
angle	814	812	811	88.0000	111.0121
angle	814	812	813	38.0000	108.9565
angle	811	812	813	60.9900	111.1250
angle	813	812	813	39.5700	108.7747
angle	812	814	821	60.0000	113.1399
angle	812	814	816	88.0000	106.2392
angle	812	814	815	38.0000	109.1733
angle	821	814	816	88.0000	110.6641
angle	821	814	815	38.0000	108.2292
angle	816	814	815	60.9900	109.3395
angle	821	816	817	88.5000	118.1296
angle	814	816	817	88.5000	119.4902
angle	819	817	816	60.0000	111.0770
angle	819	817	818	60.0000	124.9245
angle	816	817	818	50.0000	123.9986
angle	823	819	817	48.2000	113.1038
angle	823	819	820	38.0000	111.3933
angle	817	819	820	38.0000	107.4530
angle	820	819	820	45.5700	105.8127
angle	814	821	816	88.0000	108.5232
angle	814	821	822	38.0000	111.0377
angle	816	821	822	60.9900	109.5067
angle	822	821	822	39.5700	108.6681
angle	819	823	824	38.0000	107.9848
angle	823	823	824	37.9900	109.4700
angle	824	823	825	37.9900	109.3700
angle	819	823	823	48.2000	112.2989
angle	823	823	825	48.2900	112.8700
angle	824	823	824	39.6600	106.2700
angle	823	825	826	42.4200	111.2700
angle	826	825	826	39.5100	107.6500
angle	823	823	827	48.2000	112.8390
angle	823	827	827	60.0000	125.4116
angle	823	827	828	32.0000	115.6966
angle	827	827	828	32.0000	118.8892
angle	824	823	827	38.0000	108.7346
angle	823	823	823	48.2500	113.3100

```
################################
##                            ##
##   Stretch-Bend Parameters  ##
##                            ##
```

################################

strbnd	803	801	802	4.3000	14.4000
strbnd	807	803	801	18.7000	18.7000
strbnd	807	803	805	18.7000	18.7000
strbnd	807	803	804	11.5000	18.7000
strbnd	801	803	805	18.7000	18.7000
strbnd	801	803	804	11.5000	11.5000
strbnd	805	803	804	11.5000	18.7000
strbnd	803	805	806	18.7000	18.7000
strbnd	803	807	811	18.7000	18.7000
strbnd	803	807	805	11.5000	18.7000
strbnd	811	807	805	-4.5000	38.0000
strbnd	811	809	811	14.4000	14.4000
strbnd	811	809	810	14.4000	14.4000
strbnd	812	811	809	38.0000	38.0000
strbnd	807	811	809	38.0000	38.0000
strbnd	814	812	811	18.7000	18.7000
strbnd	814	812	813	11.5000	18.7000
strbnd	811	812	813	-4.5000	38.0000
strbnd	812	814	821	18.7000	18.7000
strbnd	812	814	816	18.7000	18.7000
strbnd	812	814	815	11.5000	18.7000
strbnd	821	814	816	18.7000	18.7000
strbnd	821	814	815	11.5000	18.7000
strbnd	816	814	815	-4.5000	38.0000
strbnd	821	816	817	38.0000	38.0000
strbnd	814	816	817	38.0000	38.0000
strbnd	819	817	816	18.7000	18.7000
strbnd	823	819	817	18.7000	18.7000
strbnd	823	819	820	11.5000	18.7000
strbnd	817	819	820	11.5000	18.7000
strbnd	814	821	816	18.7000	18.7000
strbnd	814	821	822	11.5000	18.7000
strbnd	816	821	822	-4.5000	38.0000
strbnd	819	823	824	11.5000	11.5000
strbnd	823	823	823	18.70	18.70
strbnd	823	823	824	11.50	18.70
strbnd	825	823	824	11.50	18.70
strbnd	819	823	823	18.70	18.70
strbnd	823	823	825	18.70	18.70
strbnd	823	825	826	11.50	11.50

#####################################
##
Out-of-Plane Bend Parameters
##
#####################################

opbend	803	805	0	0	0.2002
opbend	806	805	0	0	0.2002
opbend	816	817	0	0	0.2002
opbend	818	817	0	0	0.6500
opbend	819	817	0	0	0.2002

```
############################
##                        ##
##  Torsional Parameters  ##
##                        ##
#############################
```

torsion	802	801	803	807	0.000 0.0 1	1.000 180.0 2	0.500 0.0 3
torsion	802	801	803	805	0.000 0.0 1	1.000 180.0 2	0.500 0.0 3
torsion	802	801	803	804	0.000 0.0 1	0.661 180.0 2	0.288 0.0 3
torsion	807	803	805	806	0.649 0.0 1	3.076 180.0 2	4.783 0.0 3
torsion	801	803	805	806	-1.896 0.0 1	5.784 180.0 2	-4.176 0.0 3
torsion	804	803	805	806	0.000 0.0 1	0.000 180.0 2	0.000 0.0 3
torsion	811	807	803	801	0.000 0.0 1	-1.400 180.0 2	9.613 0.0 3
torsion	811	807	803	805	-1.717 0.0 1	0.000 180.0 2	-7.740 0.0 3
torsion	811	807	803	804	0.000 0.0 1	0.000 180.0 2	0.000 0.0 3
torsion	808	807	803	801	0.000 0.0 1	0.000 180.0 2	0.000 0.0 3
torsion	808	807	803	805	0.000 0.0 1	0.000 180.0 2	0.000 0.0 3
torsion	808	807	803	804	0.000 0.0 1	0.000 180.0 2	0.000 0.0 3
torsion	803	807	811	809	4.989 0.0 1	1.184 180.0 2	1.523 0.0 3
torsion	808	807	811	809	0.000 0.0 1	0.000 180.0 2	0.000 0.0 3
torsion	811	809	811	807	8.083 0.0 1	-1.836 180.0 2	2.637 0.0 3
torsion	810	809	811	807	0.000 0.0 1	0.000 180.0 2	0.061 0.0 3
torsion	811	809	811	812	8.083 0.0 1	-1.836 180.0 2	2.637 0.0 3
torsion	810	809	811	812	0.000 0.0 1	0.000 180.0 2	0.000 0.0 3
torsion	814	812	811	809	1.011 0.0 1	0.081 180.0 2	-0.532 0.0 3
torsion	813	812	811	809	0.000 0.0 1	0.000 180.0 2	0.000 0.0 3
torsion	821	814	812	811	-2.039 0.0 1	4.842 180.0 2	0.000 0.0 3
torsion	821	814	812	813	0.000 0.0 1	0.000 180.0 2	0.000 0.0 3
torsion	816	814	812	811	-0.776 0.0 1	1.983 180.0 2	0.000 0.0 3
torsion	816	814	812	813	0.000 0.0 1	0.000 180.0 2	0.000 0.0 3
torsion	815	814	812	811	0.000 0.0 1	0.000 180.0 2	0.000 0.0 3
torsion	815	814	812	813	0.000 0.0 1	0.000 180.0 2	0.000 0.0 3
torsion	812	814	821	816	-1.784 0.0 1	0.170 180.0 2	0.000 0.0 3
torsion	812	814	821	822	0.000 0.0 1	0.000 180.0 2	0.000 0.0 3
torsion	816	814	821	816	-2.407 0.0 1	-0.682 180.0 2	0.000 0.0 3
torsion	816	814	821	822	0.000 0.0 1	0.000 180.0 2	0.000 0.0 3
torsion	815	814	821	816	0.000 0.0 1	0.000 180.0 2	0.000 0.0 3
torsion	815	814	821	822	0.000 0.0 1	0.000 180.0 2	0.000 0.0 3
torsion	812	814	816	817	3.811 0.0 1	-1.403 180.0 2	0.000 0.0 3
torsion	821	814	816	817	4.577 0.0 1	-1.750 180.0 2	0.000 0.0 3
torsion	815	814	816	817	0.000 0.0 1	0.000 180.0 2	0.000 0.0 3

```
torsion     819   817   816   821      0.000 0.0 1    5.989 180.0 2    0.000 0.0 3
torsion     818   817   816   821      0.000 0.0 1    5.989 180.0 2    0.000 0.0 3
torsion     819   817   816   814      0.000 0.0 1    5.789 180.0 2    0.000 0.0 3
torsion     818   817   816   814      0.000 0.0 1    5.789 180.0 2    0.000 0.0 3
torsion     820   819   817   816      0.000 0.0 1    0.000 180.0 2    0.000 0.0 3
torsion     820   819   817   818      0.000 0.0 1    0.000 180.0 2    0.000 0.0 3
torsion     814   821   816   817     -0.118 0.0 1   -0.259 180.0 2   -0.230 0.0 3
torsion     822   821   816   817      0.000 0.0 1    0.000 180.0 2    0.000 0.0 3
torsion     816   817   819   823      1.231 0.0 1    0.000 180.0 2    0.000 0.0 3
torsion     818   817   819   823      0.000 0.0 1   -0.039 180.0 2    0.000 0.0 3
torsion     816   817   816   823      0.000 0.0 1   -0.039 180.0 2    0.000 0.0 3
torsion     817   819   823   823      1.532 0.0 1   -1.981 180.0 2    1.641 0.0 3
torsion     820   819   823   823      0.000 0.0 1    0.000 180.0 2    0.000 0.0 3
torsion     817   819   823   824      0.000 0.0 1    0.000 180.0 2    0.000 0.0 3
torsion     820   819   823   824      0.000 0.0 1    0.000 180.0 2    0.000 0.0 3
torsion     819   823   823   823      2.818 0.0 1   -0.410 180.0 2    2.472 0.0 3
torsion     819   823   823   824      0.000 0.0 1    0.000 180.0 2    0.000 0.0 3
torsion     823   823   823   824      0.000 0.0 1    0.000 180.0 2    0.000 0.0 3
torsion     823   823   823   823      0.568 0.0 1   -0.375 180.0 2    2.302 0.0 3
torsion     823   823   823   825      0.717 0.0 1   -0.133 180.0 2    1.123 0.0 3
torsion     824   823   823   824      0.000 0.0 1    0.000 180.0 2    0.000 0.0 3
torsion     826   825   823   823      0.000 0.0 1    0.000 180.0 2    0.341 0.0 3
torsion     826   825   823   824      0.000 0.0 1    0.000 180.0 2    0.299 0.0 3
torsion     824   823   823   825      0.000 0.0 1    0.000 180.0 2    0.000 0.0 3
torsion     823   827   827   823      0.000 0.0 1    8.000 180.0 2    0.000 0.0 3
torsion     823   823   827   827      2.775 0.0 1   -0.695 180.0 2   -1.271 0.0 3
torsion     823   827   827   828      0.000 0.0 1    6.100 180.0 2    0.000 0.0 3
torsion     828   827   827   828      0.000 0.0 1    4.070 180.0 2    0.000 0.0 3
torsion     823   823   827   828      0.000 0.0 1    0.000 180.0 2    0.000 0.0 3
torsion     824   823   823   827      0.000 0.0 1    0.000 180.0 2    0.000 0.0 3
torsion     823   823   823   827      0.649 0.0 1    0.419 180.0 2    2.472 0.0 3
torsion     824   823   827   827      0.000 0.0 1    0.000 180.0 2    0.000 0.0 3
torsion     824   823   827   828      0.000 0.0 1    0.000 180.0 2    0.000 0.0 3


      #####################################
      ##                                 ##
      ##  Atomic Multipole Parameters    ##
      ##                                 ##
      #####################################


multipole   801   803   802                0.15009
                                             0.00000    0.00000    0.39088
                                            -0.31903
                                             0.00000   -0.46883
                                             0.00000    0.00000    0.78786
```

multipole 802 801 803 0.19148
0.00205 0.00000 -0.11665
-0.12490
0.00000 0.00289
0.00403 0.00000 0.12201
multipole 803 807 801 -0.33444
0.10140 0.00000 0.26744
0.69333
0.00000 -0.87872
-0.46582 0.00000 0.18539
multipole 804 803 807 0.07315
-0.04502 0.00000 -0.15377
0.06032
0.00000 0.12156
-0.11208 0.00000 -0.18188
multipole 805 806 -806 1.09754
0.00000 0.00000 -0.06279
-0.56105
0.00000 -0.33758
0.00000 0.00000 0.89863
multipole 806 805 806 -0.80443
0.00000 0.00000 -0.07425
-0.31203
0.00000 0.04975
0.00000 0.00000 0.26228
multipole 807 803 811 0.12827
0.37502 0.00000 0.06061
0.20110
0.00000 -0.48816
-0.36209 0.00000 0.28706
multipole 808 807 803 0.02153
0.01173 0.00000 -0.07125
0.00617
0.00000 0.00959
-0.04033 0.00000 -0.01576
multipole 809 810 -810 1.75597
0.00000 0.00000 -0.03715
0.41622
0.00000 0.35763
0.00000 0.00000 -0.77385
multipole 810 809 810 -0.94589
0.00000 0.00000 -0.03105
-0.08297
0.00000 -0.26637
0.00000 0.00000 0.34934
multipole 811 807 809 -0.53707
0.01649 0.00000 0.42047
-0.42634
0.00000 -0.64972

-0.44476 0.00000 1.07606
multipole 812 813 809 -0.53707
0.05695 0.00000 0.47693
0.28714
0.00000 -0.72246
-0.19766 0.00000 0.43532
multipole 813 815 812 0.12827
0.34076 0.00000 0.07223
0.13762
0.00000 -0.55896
-0.19291 0.00000 0.42134
multipole 814 813 815 0.02153
0.03021 0.00000 -0.06371
0.05822
0.00000 -0.03614
-0.10939 0.00000 -0.02208
multipole 815 813 822 0.02757
0.12524 0.00000 0.12788
-0.17310
0.00000 0.01627
-0.12352 0.00000 0.15683
multipole 816 815 813 0.08536
-0.07809 0.00000 0.01361
0.00564
0.00000 -0.05585
-0.19930 0.00000 0.05021
multipole 817 815 818 -0.29085
0.12793 0.00000 0.43009
-0.41410
0.00000 -0.45847
-0.01045 0.00000 0.87257
multipole 818 820 817 0.78775
0.00714 0.00000 -0.23085
0.35926
0.00000 -0.09910
-0.13018 0.00000 -0.26016
multipole 819 818 820 -0.62759
0.07317 0.00000 -0.02732
-0.38754
0.00000 0.12847
0.19485 0.00000 0.25907
multipole 820 857 818 -0.15540
0.15356 0.00000 0.19795
0.12509
0.00000 -0.37752
-0.23203 0.00000 0.25243
multipole 821 820 857 0.08635
0.03876 0.00000 -0.07943
0.01560

				0.00000	0.05741	
				0.13378	0.00000	-0.07301
multipole	822	815	824	0.08475		
				0.37847	0.00000	0.14032
				0.50696		
				0.00000	-0.25857	
				-0.38564	0.00000	-0.24839
multipole	823	822	815	0.03662		
				-0.07725	0.00000	-0.09404
				-0.06019		
				0.00000	0.10374	
				-0.09173	0.00000	-0.04355
multipole	824	822	825	-0.36432		
				0.09158	0.00000	0.29562
				-0.05455		
				0.00000	-0.48997	
				-0.00432	0.00000	0.54452
multipole	825	827	824	0.96653		
				-0.14867	0.00000	0.00065
				0.62223		
				0.00000	-0.34695	
				-0.41223	0.00000	-0.27528
multipole	826	825	827	-0.67293		
				-0.07701	0.00000	-0.08391
				-0.22330		
				0.00000	0.13331	
				0.00013	0.00000	0.08999
multipole	827	829	825	-0.33708		
				-0.08126	0.00000	0.29167
				-0.14312		
				0.00000	-0.02152	
				0.23449	0.00000	0.16464
multipole	828	827	829	0.08282		
				0.07172	0.00000	-0.10755
				-0.01476		
				0.00000	0.11799	
				0.17685	0.00000	-0.10323
multipole	829	831	827	-0.12028		
				0.00000	0.00000	-0.27624
				-0.28898		
				0.00000	0.27171	
				0.00000	0.00000	0.01727
multipole	830	829	831	0.06014		
				0.02626	0.00000	0.09284
				-0.09440		
				0.00000	-0.11726	
				-0.04794	0.00000	0.21166
multipole	831	829	833	-0.12028		
				0.00000	0.00000	0.02019

0.23109
0.00000 0.51643
0.00000 0.00000 -0.74752
multipole 832 831 829 0.06014
-0.02225 0.00000 -0.03876
0.16126
0.00000 -0.12762
-0.09711 0.00000 -0.03364
multipole 833 835 831 -0.12028
0.00000 0.00000 0.38294
-0.23632
0.00000 0.09598
0.00000 0.00000 0.14034
multipole 834 833 835 0.06014
-0.05338 0.00000 -0.05236
0.05217
0.00000 -0.03071
-0.00241 0.00000 -0.02146
multipole 835 833 837 -0.12028
0.00000 0.00000 0.08043
-0.28788
0.00000 0.15240
0.00000 0.00000 0.13548
multipole 836 835 833 0.06014
0.01189 0.00000 0.06548
-0.04124
0.00000 -0.12950
0.02846 0.00000 0.17074
multipole 837 839 835 -0.12028
0.00000 0.00000 0.19119
-0.24827
0.00000 0.18257
0.00000 0.00000 0.06570
multipole 838 837 839 0.06014
-0.01631 0.00000 0.07167
-0.05599
0.00000 -0.10649
-0.00219 0.00000 0.16248
multipole 839 837 -841 -0.12028
0.00000 0.00000 0.16276
0.40325
0.00000 -0.24444
0.00000 0.00000 -0.15881
multipole 840 839 841 0.06014
0.01220 0.00000 0.06285
-0.12021
0.00000 -0.07078
0.02300 0.00000 0.19099
multipole 841 843 839 -0.12028

0.00000 0.00000 -0.00371
-0.27290
0.00000 0.24262
0.00000 0.00000 0.03028
multipole 842 841 839 0.06014
0.00111 0.00000 0.17584
-0.11309
0.00000 -0.19933
0.02464 0.00000 0.31242
multipole 843 845 841 -0.12028
0.00000 0.00000 -0.03035
-0.38338
0.00000 -0.11244
0.00000 0.00000 0.49582
multipole 844 843 845 0.06014
0.10649 0.00000 0.17604
-0.15014
0.00000 -0.17048
0.15885 0.00000 0.32062
multipole 845 843 -847 -0.12028
0.00000 0.00000 0.21358
0.41809
0.00000 -0.33917
0.00000 0.00000 -0.07892
multipole 846 845 847 0.06014
-0.01053 0.00000 0.17164
-0.21578
0.00000 -0.14027
-0.02295 0.00000 0.35605
multipole 847 849 845 -0.12028
0.00000 0.00000 0.08114
-0.43635
0.00000 0.13193
0.00000 0.00000 0.30442
multipole 848 847 849 0.06014
0.01274 0.00000 0.26178
-0.22740
0.00000 -0.24814
0.04916 0.00000 0.47554
multipole 849 847 851 -0.12028
0.00000 0.00000 0.28031
0.20634
0.00000 -0.50100
0.00000 0.00000 0.29466
multipole 850 849 851 0.06014
0.04950 0.00000 0.14333
-0.14948
0.00000 -0.16464
-0.00394 0.00000 0.31412

multipole 851 849 -853 -0.12028
0.00000 0.00000 0.10812
0.17407
0.00000 0.10057
0.00000 0.00000 -0.27464
multipole 852 851 853 0.06014
-0.00763 0.00000 0.14643
-0.13955
0.00000 -0.13611
0.00017 0.00000 0.27566
multipole 853 851 855 -0.12028
0.25888 0.00000 -0.07373
-0.40444
0.00000 0.18904
-0.17448 0.00000 0.21540
multipole 854 853 855 0.06014
-0.11707 0.00000 0.13841
-0.14215
0.00000 -0.15682
-0.13312 0.00000 0.29897
multipole 855 853 856 -0.16638
0.00000 0.00000 0.17570
-0.11035
0.00000 -0.08951
0.00000 0.00000 0.19986
multipole 856 855 853 0.05546
0.00839 0.00000 0.05163
-0.07452
0.00000 -0.10058
0.02040 0.00000 0.17510
multipole 857 859 820 -0.12028
0.00000 0.00000 0.16483
-0.34157
0.00000 0.29032
0.00000 0.00000 0.05125
multipole 858 857 859 0.06014
-0.09385 0.00000 0.06163
-0.05752
0.00000 -0.11452
-0.16015 0.00000 0.17204
multipole 859 861 857 -0.12028
0.00000 0.00000 0.21055
0.06973
0.00000 -0.16737
0.00000 0.00000 0.09764
multipole 860 859 857 0.06014
0.11641 0.00000 -0.06467
0.17364
0.00000 -0.07819

0.11793 0.00000 -0.09545
multipole 861 863 859 -0.12028
0.00000 0.00000 0.22207
-0.45142
0.00000 0.12922
0.00000 0.00000 0.32220
multipole 862 861 863 0.06014
-0.05716 0.00000 0.04611
-0.08401
0.00000 -0.02608
-0.12239 0.00000 0.11009
multipole 863 861 -865 -0.12028
0.00000 0.00000 0.24675
0.81584
0.00000 -0.41153
0.00000 0.00000 -0.40431
multipole 864 863 865 0.06014
0.00068 0.00000 -0.06235
0.06236
0.00000 0.04198
0.01631 0.00000 -0.10434
multipole 865 863 867 -0.12028
0.00000 0.00000 0.26583
-0.29207
0.00000 -0.09693
0.00000 0.00000 0.38900
multipole 866 865 863 0.06014
-0.01064 0.00000 -0.01444
-0.04530
0.00000 -0.02924
-0.01287 0.00000 0.07454
multipole 867 865 869 -0.08684
0.16232 0.00000 0.16012
0.20689
0.00000 0.04491
-0.27071 0.00000 -0.25180
multipole 868 867 865 0.06914
-0.02374 0.00000 -0.10784
0.13103
0.00000 0.01169
0.00244 0.00000 -0.14272
multipole 869 867 871 -0.10494
0.01927 0.00000 0.13175
-0.00325
0.00000 -0.10324
0.23581 0.00000 0.10649
multipole 870 869 867 0.05350
0.01400 0.00000 -0.09671
0.11469

0.00000 -0.06088
-0.00053 0.00000 -0.05381
multipole 871 873 869 -0.10494
0.01803 0.00000 0.12881
0.07379
0.00000 -0.16541
0.22004 0.00000 0.09162
multipole 872 871 873 0.05350
0.01945 0.00000 -0.10314
0.15986
0.00000 -0.05584
0.01670 0.00000 -0.10402
multipole 873 875 871 -0.08684
0.15984 0.00000 0.15576
0.15285
0.00000 -0.00851
-0.33188 0.00000 -0.14434
multipole 874 873 875 0.06914
-0.02413 0.00000 -0.11505
0.10858
0.00000 0.03784
-0.01793 0.00000 -0.14642
multipole 875 877 873 -0.12028
0.00000 0.00000 0.26690
-0.09146
0.00000 0.03942
0.00000 0.00000 0.05204
multipole 876 875 877 0.06014
-0.02429 0.00000 -0.00551
-0.01934
0.00000 -0.04490
-0.01314 0.00000 0.06424
multipole 877 875 -879 -0.12028
0.00000 0.00000 0.26491
0.48080
0.00000 -0.40857
0.00000 0.00000 -0.07223
multipole 878 877 879 0.06014
0.00166 0.00000 -0.04633
0.02742
0.00000 0.02210
-0.03703 0.00000 -0.04952
multipole 879 877 881 -0.12028
0.00000 0.00000 0.25521
-0.24375
0.00000 0.21673
0.00000 0.00000 0.02702
multipole 880 879 877 0.06014
-0.04480 0.00000 0.03903

```
                                    -0.05620
                                     0.00000   -0.05569
                                    -0.04031    0.00000    0.11189
multipole   881  879  883           -0.12028
                                     0.00000    0.00000    0.20623
                                     0.13103
                                     0.00000   -0.20157
                                     0.00000    0.00000    0.07054
multipole   882  881  883            0.06014
                                     0.12155    0.00000   -0.09278
                                     0.12585
                                     0.00000    0.02360
                                     0.18633    0.00000   -0.14945
multipole   883  881 -885           -0.12028
                                     0.00000    0.00000    0.25000
                                     0.29752
                                     0.00000   -0.37898
                                     0.00000    0.00000    0.08146
multipole   884  883  885            0.06014
                                     0.02573    0.00000   -0.10674
                                     0.10417
                                     0.00000    0.02370
                                     0.02516    0.00000   -0.12787
multipole   885  883  887           -0.12028
                                     0.30410    0.00000    0.17140
                                     0.08329
                                     0.00000   -0.50724
                                    -0.27934    0.00000    0.42395
multipole   886  885  887            0.06014
                                     0.02307    0.00000   -0.14797
                                     0.11998
                                     0.00000    0.08791
                                     0.03663    0.00000   -0.20789
multipole   887  885  888           -0.16638
                                     0.00000    0.00000    0.25061
                                    -0.29507
                                     0.00000   -0.32451
                                     0.00000    0.00000    0.61958
multipole   888  887  885            0.05546
                                     0.00780    0.00000   -0.07615
                                    -0.01142
                                     0.00000    0.03391
                                    -0.02243    0.00000   -0.02249


      ########################################
      ##                                    ##
```

Dipole Polarizability Parameters
##
##

polarize	801	1.073	0.390	802 803
polarize	802	0.496	0.390	801
polarize	803	1.334	0.390	801 804 805
polarize	804	0.496	0.390	803
polarize	805	1.334	0.390	803 806
polarize	806	0.921	0.390	805
polarize	807	1.334	0.390	808 811
polarize	808	0.496	0.390	807
polarize	809	1.828	0.390	810 811 812
polarize	810	0.837	0.390	809
polarize	811	0.837	0.390	807 809
polarize	812	0.837	0.390	809 813
polarize	813	1.334	0.390	812 814
polarize	814	0.496	0.390	813
polarize	815	1.334	0.390	816 817
polarize	816	0.496	0.390	815
polarize	817	0.837	0.390	815 818
polarize	818	1.334	0.390	817 819 820
polarize	819	0.837	0.390	818
polarize	820	1.334	0.390	818 821
polarize	821	0.496	0.390	820
polarize	822	1.334	0.390	823 824
polarize	823	0.496	0.390	822
polarize	824	0.837	0.390	822 825
polarize	825	1.334	0.390	824 826 827
polarize	826	0.837	0.390	825
polarize	827	1.334	0.390	825 828
polarize	828	0.496	0.390	827
polarize	829	1.334	0.390	830 831
polarize	830	0.496	0.390	829
polarize	831	1.334	0.390	829 832
polarize	832	0.496	0.390	831
polarize	833	1.334	0.390	834 835
polarize	834	0.496	0.390	833
polarize	835	1.334	0.390	833 836
polarize	836	0.496	0.390	835
polarize	837	1.334	0.390	838 839
polarize	838	0.496	0.390	837
polarize	839	1.334	0.390	837 840
polarize	840	0.496	0.390	839
polarize	841	1.334	0.390	842 843
polarize	842	0.496	0.390	841
polarize	843	1.334	0.390	841 844
polarize	844	0.496	0.390	843

polarize	845	1.334	0.390	846	
polarize	846	0.496	0.390	845	
polarize	847	1.334	0.390	848 849	
polarize	848	0.496	0.390	847	
polarize	849	1.334	0.390	847 850	
polarize	850	0.496	0.390	849	
polarize	851	1.334	0.390	852	
polarize	852	0.496	0.390	851	
polarize	853	1.334	0.390	854 855	
polarize	854	0.496	0.390	853	
polarize	855	1.334	0.390	853 856	
polarize	856	0.496	0.390	855	
polarize	857	1.334	0.390	858	
polarize	858	0.496	0.390	857	
polarize	859	1.334	0.390	860	861
polarize	860	0.496	0.390	859	
polarize	861	1.334	0.390	859	862
polarize	862	0.496	0.390	861	
polarize	863	1.334	0.390	864	865
polarize	864	0.496	0.390	863	
polarize	865	1.334	0.390	863	866
polarize	866	0.496	0.390	865	
polarize	867	1.334	0.390	868	
polarize	868	0.496	0.390	867	
polarize	869	1.334	0.390	870	871
polarize	870	0.496	0.390	869	
polarize	871	1.334	0.390	869	872
polarize	872	0.496	0.390	871	
polarize	873	1.334	0.390	874	
polarize	874	0.496	0.390	873	
polarize	875	1.334	0.390	876 877	
polarize	876	0.496	0.390	875	
polarize	877	1.334	0.390	875 878	
polarize	878	0.496	0.390	877	
polarize	879	1.334	0.390	880 881	
polarize	880	0.496	0.390	879	
polarize	881	1.334	0.390	879 882	
polarize	882	0.496	0.390	881	
polarize	883	1.334	0.390	884	
polarize	884	0.496	0.390	883	
polarize	885	1.334	0.390	886 887	
polarize	886	0.496	0.390	885	
polarize	887	1.334	0.390	885 888	
polarize	888	0.496	0.390	887	
